# Heterogeneous responses of temperate-zone amphibian populations to climate change complicates conservation planning

**DOI:** 10.1038/s41598-017-17105-7

**Published:** 2017-12-06

**Authors:** E. Muths, T. Chambert, B. R. Schmidt, D. A. W. Miller, B. R. Hossack, P. Joly, O. Grolet, D. M. Green, D. S. Pilliod, M. Cheylan, R. N. Fisher, R. M. McCaffery, M. J. Adams, W. J. Palen, J. W. Arntzen, J. Garwood, G. Fellers, J.-M. Thirion, A. Besnard, E. H. Campbell Grant

**Affiliations:** 1 U.S. Geological Survey, Fort Collins Science Center, 2150 Centre Ave., Bldg C, Fort Collins, CO 80526 USA; 2 Pennsylvania State University, Department of Ecosystem Science and Management, University Park, PA 16802 USA; 3 U.S. Geological Survey, Patuxent Wildlife Research Center, Laurel, MD 20708 USA; 4 Department of Evolutionary Biology and Environmental Studies, University of Zurich, 8057 Zurich, Switzerland; 5 Info Fauna KARCH, 2000 Neuchâtel, Switzerland; 6 U.S. Geological Survey, Aldo Leopold Wilderness Research Institute, 790 E. Beckwith, Missoula, MT 59801 USA; 7 Université Lyon 1, UMR 5023 - LEHNA, Laboratoire d’Ecologie des Hydrosystèmes Naturels et Anthropisés, 69100 Villeurbanne, France; 8 Redpath Museum, McGill University, 859 Sherbrooke St. W. Montreal, Quebec, H3A 2K6 Canada; 9 U.S. Geological, Survey Forest and Rangeland Ecosystem Science Center, 970 Lusk St, Boise, ID 83706 USA; 10 CNRS, PSL Research University, EPHE, UM, SupAgro, IRD, INRA, UMR 5175 CEFE, F-34293, Montpellier, France; 11 U.S. Geological Survey, Western Ecological Research Center, San Diego Field Station, 4165 Spruance Road, San Diego, CA 92101 USA; 12 University of Montana, Division of Biological Sciences, 32 Campus Dr., Missoula, MT USA; 13 U.S. Geological Survey, Forest and Rangeland Ecosystem Science Center, 3200 SW Jefferson Way, Corvallis, OR 97331 USA; 14 Simon Fraser University, Department of Biological Sciences, 8888 University Drive Burnaby, British Columbia, CANADA V5A 1S6 Canada; 15 Naturalis Biodiversity Center, 6.4.16 Sylvius Bldg, 2333 CR Leiden, The Netherlands; 16 California Department of Fish and Wildlife, 5341 Ericson Way, Arcata, CA 95521 USA; 17 U.S. Geological Survey, Western Ecological Research Center, Point Reyes National Seashore, Point Reyes, CA 94956 USA; 18 Association Objectifs Biodiversités (OBIOS), 12 rue du docteur Gilbert, 17250 Pont l’Abbé d’Arnoult, France; 19 U.S. Geological Survey, Patuxent Wildlife Research Center, SO Conte Anadromous Fish Laboratory, One Migratory Way, Turners Falls, MA 01376 USA; 20 Present Address: U.S. Geological Survey, Forest and Rangeland Ecosystem Science Center, 600 E. Park Ave, Port Angeles, WA 98362 USA

## Abstract

The pervasive and unabated nature of global amphibian declines suggests common demographic responses to a given driver, and quantification of major drivers and responses could inform broad-scale conservation actions. We explored the influence of climate on demographic parameters (i.e., changes in the probabilities of survival and recruitment) using 31 datasets from temperate zone amphibian populations (North America and Europe) with more than a decade of observations each. There was evidence for an influence of climate on population demographic rates, but the direction and magnitude of responses to climate drivers was highly variable among taxa and among populations within taxa. These results reveal that climate drivers interact with variation in life-history traits and population-specific attributes resulting in a diversity of responses. This heterogeneity complicates the identification of conservation ‘rules of thumb’ for these taxa, and supports the notion of local focus as the most effective approach to overcome global-scale conservation challenges.

## Introduction

### Amphibian decline | demography | populations | climate

The response of biodiversity to climate change is often examined at macroecological and temporal scales, treating demographic mechanisms implicitly (e.g.,^1^, but see^2^). Demographic rates describe changes in abundance and distribution for populations, but reflect abilities of individual animals to respond to changing conditions and local environments. This implies that environmental variation is a powerful determinant of natural selection and ultimately determines individual fitness, and the dynamics and viability of populations^3,4^. Thus, a better understanding of the extent to which climate forces changes to population demographic rates, and how this affects the synchronicity of population responses to large-scale environmental fluctuations is critical to understanding the scope of potential losses, predicting future population viability, and developing strategies to preserve biodiversity^5–7^. For example, if a common, and predictable, demographic response (e.g., increasing or decreasing synchrony) to climate change could be identified, global conservation strategies, or “rules of thumb” might also be identified to combat declines.

While large-scale climate conditions can synchronize population dynamics, local environmental conditions often mediate the effects of regional drivers, such that local populations vary asynchronously and are thus more resilient to large-scale climate variation (e.g.,^8^). Conversely, if demographic rates of populations are synchronized by large-scale environmental forcing, then adverse change in regional conditions may lead to large-scale population declines. Synchronous responses to climate drivers have been identified for populations of different species over large regions^1,2,9^, suggesting that the potential exists for a single dominant driver to force synchrony in population dynamics by affecting one or more demographic rates. Furthermore, there is evidence that climatic fluctuations contribute to shifts in demographic processes of populations^10–13^, and that climate-mediated declines in animal populations^5,14^ occur. The geographic extent of amphibian decline contributes to the appeal of this phenomenon as a model system to understand the effects of climate on populations and persistence, and widespread declines in amphibian populations^15^ suggest that a single dominant driver may be forcing regional synchrony in amphibian population dynamics by affecting one or more demographic rates.

Demographic rates that characterize the response of animals to change may respond to variation occurring at two scales^16^: landscape, where over-arching processes produce similarity in demographic rates (e.g., alignment across populations^17,18^, and local, where over-arching processes manifest differently and produce differences in demographic rates (e.g., increased local variation). Directional changes in climate, such as long-term shifts in mean precipitation and timing, can act at a landscape scale and influence populations across a large area^19^, whereas outcomes of such shifts (e.g., amount of snowpack or hydroperiod length) can act at local scales and affect only individual populations because the shifts are mediated by other factors that vary locally (e.g., soil type). For example, a broad scale effect is illustrated by the link between climate change and the collapse of population cycles^20^, and a local effect is illustrated by the influence of snow depth as a consequence of the weather on the synchrony in population growth rates^21^.

While climate can influence vertebrate populations at large scales^22^, most taxa evaluated have either large ranges or are migratory. Amphibians have relatively small ranges and may not track climate changes as well as more mobile taxa^23^, and thus be more sensitive. Climate change is expected to affect both mean and variance of environmental temperature and water availability^24,25^, and this shift is accelerating^26^. As ectotherms with permeable skin, amphibians are sensitive to environmental temperature and moisture^27^, and we expect that demographic rates in amphibians are especially sensitive to a changing climate, especially to increases in the frequency or intensity of extreme conditions^28–30^. Typically, local conditions, unrelated to weather, buffer against climatic fluctuations. However, the current magnitude and rate of climate change suggest that forcing of regional water availability (i.e., drought) and temperature has the potential to over-ride local variation in these characteristics, leading to an alignment in among-population demographic rates. For example, many temperate amphibian species exist as metapopulations^31^, but demography in populations fluctuates in response to local conditions^11,32^, suggesting that large metapopulations are maintained when regional synchrony (due to broad-scale environmental variation) is low.

Current knowledge about the response of amphibian populations to climate is based largely on analyses of single-population datasets. Furthermore, when large-scale drivers are filtered through local site and population characteristics^8^, it is difficult to identify common factors driving demographic trends. The identification of common population responses to changes in climate can be improved by articulating and testing hypotheses about responses of demographic rates to environmental covariates using long-term data from multiple species within single taxa. This information will improve understanding of the relative contribution of regional and local drivers which are critical for estimating adaptive capacity and choosing conservation actions.

**Figure 1:**
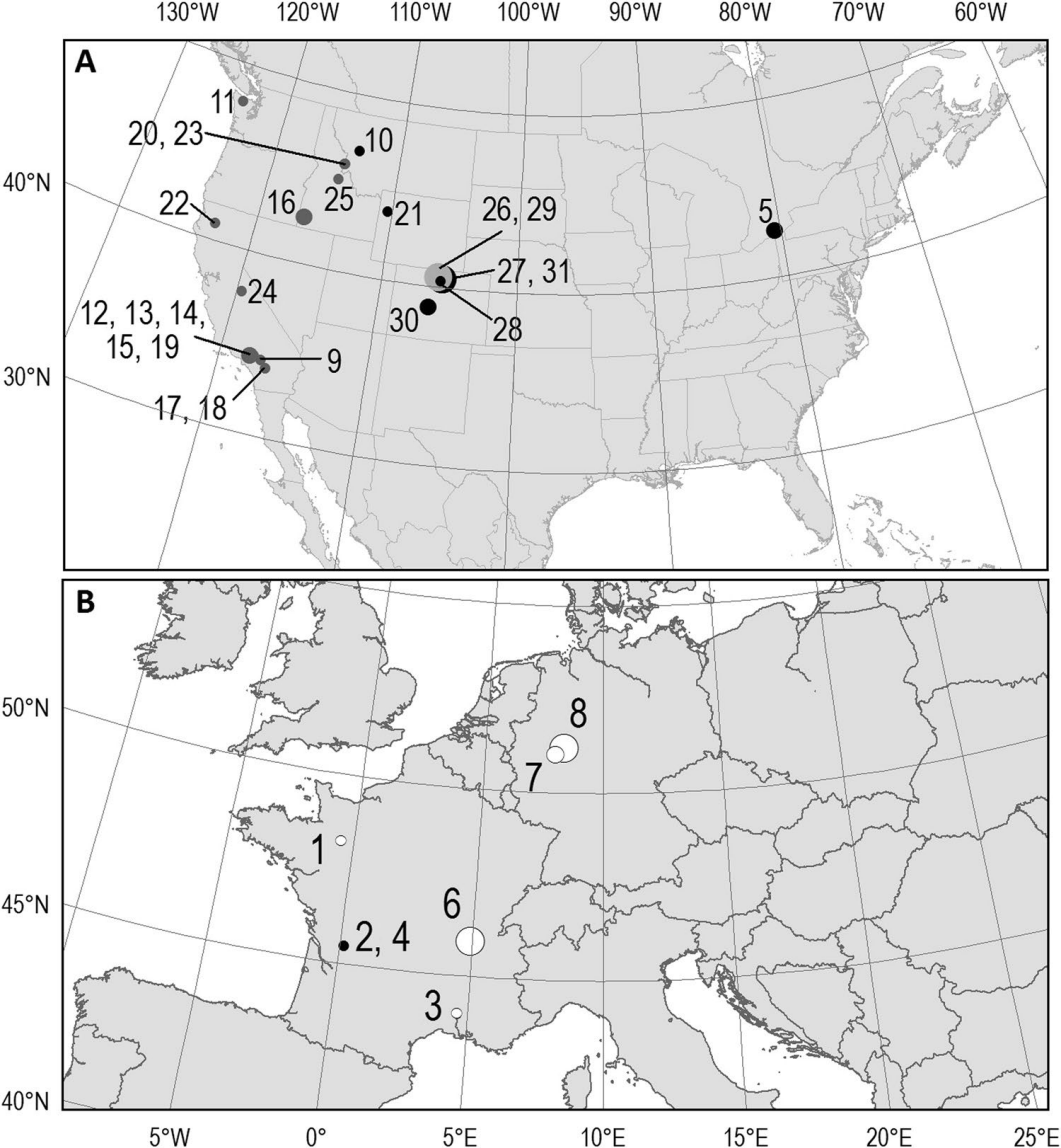
Location of study sites. (**A**) North America, (**B)** Europe. Numbers correspond to data sets (S1). Black = bufonids; light grey = treefrogs; dark grey = ranids; white = salamanders and newts. Size of dot represents length of data set: large >20 yrs (n = 6); medium = 15–20 yrs (n = 5); small = 10–14 yrs (n = 20). Map produced using ArcMap Version 10.3.

Based on evidence of the substantial magnitude and increasing rate of climate change^24–26^, and the knowledge that weather affects demographic rates^28,33–35^, we quantified survival and recruitment in relation to locally derived climate variables to gain a mechanistic understanding of amphibian demographic responses across a range of species and locations. We used long-term (≥10 or more consecutive years), individual-based, information from 31 populations of amphibians from four countries in Europe and North America representing three climatic “zones”: montane, Mediterranean, and maritime (Canada [n = 1], France [n = 5], Germany [n = 2], and USA [n = 23]) (Fig. 1 and S1) with which to estimate the effect of climate forcing on demography. Capture-mark-recapture data are uniquely rich and provide the most detailed information on free-ranging populations. Our depth of data allows consideration of hypotheses that relate demographic parameters to broad-scale climate, as represented by specific local climate variables. We selected covariates representing local environmental conditions that are driven by broad-scale climatic changes in temperature and water availability. We predicted demographic responses in a suite of *a priori* hypotheses (Table 1) iteratively developed by expert amphibian researchers with specific knowledge of local populations and habitats – collectively representing >300 years of field expertise. We expected that the demography of species with similar life histories (i.e., terrestrial vs aquatic hibernation), and those inhabiting similar environments (i.e., maritime, Mediterranean, and montane zones) would have similar associations with climate drivers. We also expected that survival, recruitment (i.e., the per capita number of individuals recruited into the breeding populations per year) and population growth rate (i.e., survival + recruitment) might each be influenced in a common manner across populations of amphibian species when they experience similar changes in abiotic conditions. While the magnitude of effect is not expected to be identical among all species and populations, we expected that the direction of the effect would be consistent. Although commonalities among demographic rates or characteristics can exist without synchrony in environmental conditions, synchronous relationships may be an early indicator of population collapse as the buffering that diversity provides is weakened.

**Table 1:** To assess survival, we hypothesized that: 1) adult survival is reduced by lack of water during active non-breeding season (H1) because of potential for physiological stress (Bartelt *et al*. 2004), lower food availability (Williams 1951) and general habitat degradation (pond drying, Amburgey *et al*. 2012, Hossack *et al*.^37^, Pilliod and Scherer 2015); 2) Unusually high temperatures can influence survival negatively due to desiccation and related lack of water (Rittenhouse *et al*. 2015) (H2); 3) Longer winters decrease time available for an individual to be active and reduce survival (e.g., emerging from long hibernation in weakened state; or reduced opportunities for foraging) (Carey *et al*. 2005,) (H3); 4) In montane habitats, exposure to cold temperatures reduce survival (H4). However, cold experienced by hibernating animals is buffered by snowpack; in low snowpack years, cold temperatures may have greater impact. Thus, snowpack (as represented by a measure of snow water equivalent [SWE]) was accounted for in developing covariate H4 (O’Connor and Rittenhouse 2016); 5) Bouts of unseasonably warm temperatures during hibernation (i.e., winter) cause physiological arousal (Sinclair *et al*.^40^), interrupt hibernation, waste energy, and reduce survival (H5). Snowpack also buffers against warm temperatures so we accounted for the effect of snowpack during the bouts of warm weather in winter. To assess recruitment we hypothesized that: 1) Recruitment is reduced by lack of water due to increased desiccation risk to small bodied juveniles, and reduced food and habitat (H6); 2) Longer and colder winters decrease the amount of time available for an individual to be active and will reduce survival and thus recruitment (H7); 3) Cold temperatures in spring that result in damage or destruction of eggs will reduce recruitment (Håkansson and Loman 2004) (H8); and 4) Freezing events in the autumn before metamorphic animals have successfully left the breeding site will reduce recruitment; in other words, if the onset of winter (i.e., freezing events) is later, recruitment will be influenced positively (H9).

	Hypothesis	Species	Prediction	Season	Covariate and Formulation*	Mechanism	Mathematical model
Survival	H1	All	Survival decreases in drier conditions	Active non-breeding season - breeding activity in spring through the onset of hibernation	DROUGHT: Number of drought days (i.e., no precipitation) during the active, non-breeding season in a given year. Drought “events” were categorized as groups of days interspersed by days with rain. Drought days were counted after a site-specific threshold number of consecutive drought days was reached for each “event”. Thresholds were defined relative to the mean climate at the site and were chosen such that the coefficient of variation was >0.95 for that covariate.	Desiccation, less food, fewer hibernacula	logit(survival) = B0 + B1 * CovH1
	H2	All	Survival decreases as the number of unusual warm days increase	Active season - emergence from hibernation to the onset of hibernation.	UNUSUAL WARM DAYS: Number of warm days during the active season. Warm is defined as a day with a maximum temperature at least 2 SD above the average 30-year maximum temperature.	Heat Stress	logit(survival) = B0 + B2 * CovH2
	H3	All	Survival decreases as the length of winter increases	Winter	LENGTH OF WINTER: The ratio of winter length relative to length of (previous) active (breeding and non-breeding) season. Winter is defined as the period between the first and last killing frost (−4.44 °C or 0 °C for warm regions).	Energy expenditure during hibernation	logit(survival) = B0 + B3 * CovH3
	H4	Terrstrial hibernators	Survival decreases as winter severity increases	Winter	WINTER SEVERITY: Number of cold days during winter. Cold defined as minimum temperature at least 2 SD below the 30 year average minimum temperature. Cold “events” categorized as groups of cold days interspersed by warmer days. Cold days counted after a site-specific threshold number of consecutive cold days was reached for each “event”. Thresholds for cold events defined relative to the mean climate at the site and chosen such that the coefficient of variation was >0.95 for that covariate.	Freezing (when exposed to cold temperatures)	logit(survival) = B0 + B4 * CovH4
	H5	All	Survival decreases as warm days during hibernation increase	Middle winter: Defined as the period between the 10 and 90% quantile of winter length.	WARM DAYS DURING HIBERNATION: Number of unusually warm days during middle of winter. Warm is defined as mean maximum temperature at least 2 SD above the 30 year mean maximum temperature. Warm “events” categorized as groups of warm days interspersed by cooler days. Warm days counted after a site-specific threshold number of consecutive warm days was reached for each “event”. Thresholds for warm events defined relative to the mean climate at the site and chosen such that the coefficient of variation was >0.95 for that covariate.	Inappropriate Rousing - Waste of energy	logit(survival) = B0 + B5 * Cov-H5
Recruitment	H6	All	Recruitment decreases with the increase in drought conditions	Active non-breeding season from year t-LAG to year t	DROUGHT: Number of drought days (i.e., no precipitation) during the active, non-breeding season cumulated from year t-LAG to year t.	Desiccation, less food, fewer hibernacula during juvenile years	log(recruitment) = B0 + B6 * CovH6
	H7	All	Recruitment decreases with longer winters over juvenile years	Winter	LENGTH OF WINTER: Ratio of winter length relative to length of (previous) active season, cumulated from year t-LAG to year t. Winter defined as the period between first and last killing frost (−4.44 °C).	Hibernation energy expenditure, energy storage & hypoxia	log(recruitment) = B0 + B7 * CovH7
	H8	All	Recruitment decreases with increased freezing events during egg laying	Egg laying period (2 wks before - 2 wks after the approximate date of egg-laying (S1).	COLD TEMPERATURES: Number of cold days during the egg laying period in year t-LAG. Cold is defined as a day with an average minimum temperature at least 2 SD below the 30 year average minimum temperature.	Freezing of eggs	log(recruitment) = B0 + B8 * CovH8
	H9	All	Recruitment increases with later winter onset	Autumn	DATE OF WINTER ONSET: Date of first sustained killing frost in year of metamorphosis. Killing frost defined as temperature <−4.44 °C or <0 °C for warm regions; sustained defined as ≥3 consecutive days.	Freezing before metmorphosis	log(recruitment) = B0 + B1 * CovH9

*Refer to Supporting information (S7) for covariate values and sources.

## Results

We analyzed data from 11 species and 31 populations where individuals were captured annually from 10 to 28 years (average = 15.3 yrs). We found evidence for an influence of climate on demography (Akaike weight >0.40, 18/31 datasets), although the magnitude and direction of responses to climate drivers was highly variable. Capture probabilities ranged from <0.01–0.74, mean 0.16 (S2). Survival and recruitment estimates were within published norms for all species, taxa were not evenly distributed among climate zones (5 species in maritime, 2 in Mediterranean, and 5 in montane) and population sizes varied. For example, in the Mediterranean zone, 8 of 9 datasets were from small populations of ranid frogs in California.

### Adult survival

**Figure 2:**
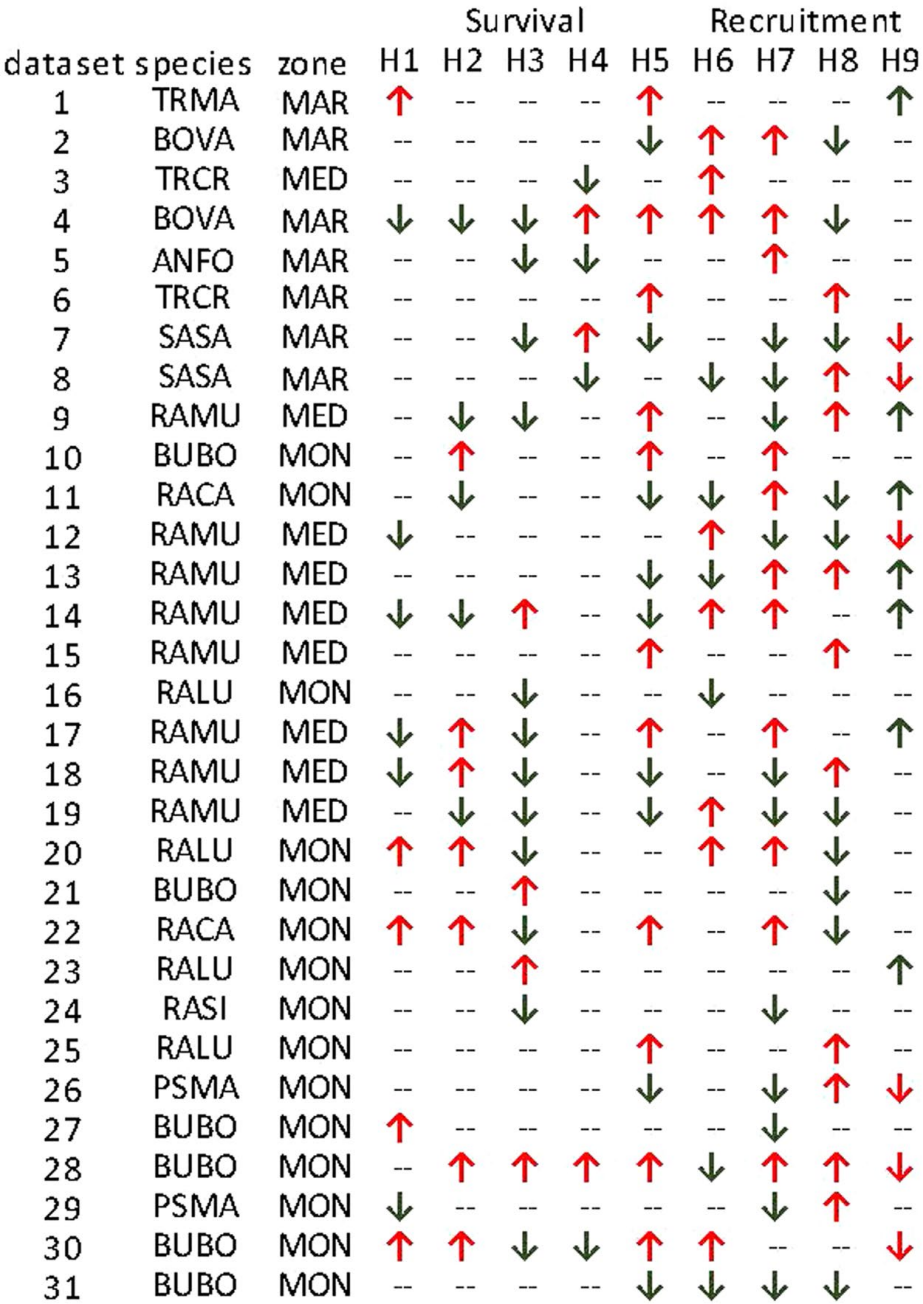
Hypotheses supported by models (based on the covariate [log-odds] coefficient parameter) are indicated by an arrow. The predicted effect for hypotheses H1 – H8 was negative, but the predicted effect for H9 was positive. Direction of arrow indicates direction of support (H1-H8: down = as predicted, up = contrary to prediction; H9: up = as predicted, and down = contrary to prediction). Data sets are ordered from low to high elevation. See S1 for details on datasets and species names and Table 1 for hypotheses. Zone: MAR – maritime, MED – Mediterranean, MON – montane.

No single hypothesis was strongly supported across all datasets; models including each of our hypothesized drivers were the best supported in at least one case (Fig. 2, S3 and S4). While most of the sites in the Mediterranean zone (8/9) followed predictions for the hypothesized direction of the effect, only about half of the maritime (4/7) and montane (7/15) sites followed our predictions. For most sites (23/31), top models for survival included winter-related covariates (H3, H4, H5). QAICc weights and r^2^ values (S3, S5) indicated that models that included the length of winter (H3) and winter severity (H4) were often best supported. Covariates in top models more often followed the predicted direction for frogs (13/17 populations) and salamanders and newts (3/5 populations) than for toads (3/9 populations). The direction of the effect of the covariate in the top model was more often as predicted for aquatic hibernators (11/15) than for terrestrial hibernators (8/16).

### Recruitment

Similarly, no single recruitment hypothesis received overwhelming support across datasets (see Methods for definition). Each of the hypothesized drivers were in the top model for at least one amphibian population (Figs 2, S4 and S5). Top models for recruitment also included winter-related covariates (H7, H8, H9) for 24 of 31 sites. Model results were consistent with predictions 39% of the time (13/31 datasets). The direction of even well-supported effects sometimes differed among sites. For example, cold temperature (H8) was in the top model for 11/31 data sets but the direction of the effect at 7/11 sites was contrary to our prediction of decreased recruitment with increased freezing events. Other covariates appeared in top models but results were split evenly between the direction of the effect supporting or countering our predictions. Parameter estimates (model-averaged across all sites) indicated the most support for models that included drought (H6, β = 0.001, SE = 3.60E-05) and early winter (H9, β = 0.001, SE = 4.60E-05) (S3 and S4). The covariates in the top models for recruitment were poor predictors of the hypothesized effect (i.e., they had weak estimated effect and high uncertainty) in all zones, although half of the top models in the montane zone followed our predictions. *A posteriori* examination of the results indicated that the correspondence of estimated and predicted direction of effect was not related to taxon.

### Variation

The influence of climate on demography was best illustrated in the variance analyses. The most supported models for survival explained over 25% of the temporal deviance (aka variation) in 19/31 datasets (12/19 where model results were as predicted). The best supported models for recruitment explained over 25% of the variation in 16/31 datasets (6/16 where model results were as predicted) (S5).

#### By zone

In top models for survival, where the effect was in the predicted direction, hypotheses explained 11–44% of the variation in survival for amphibians in the maritime zone; 10–69% of the variation in survival in the Mediterranean zone; and 10–55% of the variation in survival in the Montane zone (S5). In top recruitment models where the effect was in the predicted direction, the amount of variation explained was 19–41% maritime; 50–53% Mediterranean; and 6–46% montane (S5).

#### By taxon

In top models for survival, where the effect was in the predicted direction, hypotheses explained 10–69% of the variation for survival in frogs (ranids and treefrogs), 11–46% of the variation for survival in toads, and 10–44% of the variation in newts (S6B). In top recruitment models where the effect was in the predicted direction, the amount of variation explained was 6–53% for frogs (ranids and treefrogs), 8–46% for toads, and 19–41% for newts (S5).

#### By hibernation mode

In top models for survival, where the effect was in the predicted direction, hypotheses explained 10–48% of the variation for survival in amphibians that hibernate terrestrially and 19–69% of the variation for survival in amphibians that hibernate aquatically (S5). In top recruitment models where the effect was in the predicted direction, the amount of variation explained was 8–46% for terrestrial hibernators and 6–53% for aquatic hibernators (S5).

## Discussion

We provide empirical evidence for the idea that climate is one of the overarching drivers for change in amphibian demographics and that demographic parameters are influenced by climate-related covariates for amphibian populations in multiple temperate climates. Although there are numerous single species (or single locale) studies that examine how amphibians respond to their environment^36,37^, and link climate to changes in demography^14,29,34,35,38^, we capitalized on the greater taxonomic, spatial and temporal scales of our data, and hypotheses formulated on theory, empirical evidence, and expert elicitation (i.e., our collective field experience), to assess the potential for common climate-driven responses of multiple species across large geographic regions.

Our analyses of 31 long-term demographic datasets revealed variable demographic responses to climate variables both within and across species, and within and across climatic zones. In 18 of 31 datasets, the top candidate models were strongly supported with AIC_c_ wts of >0.40; in nine of those 18, AIC_c_ wts were >0.90. We considered this to be strong support because 0.4 is 10 times greater than the AIC_c_ wt one would expect if all models were equally supported (i.e., for each dataset we developed 30 models for survival and 25 for recruitment. If all models had equal support, we would expect an AIC_c_ wt of 0.04 (1/25) or 0.03 (1/30) respectively. Thus, our analysis provides empirical support for the effect of climate variables on demographic processes across multiple amphibian taxa and across multiple localities. The influence of the covariates was also supported by the amount of variation explained by the hypotheses. In a majority of datasets, ≥25% of the variation was explained for both survival and recruitment, further highlighting the importance of climate.

While no single covariate emerged as a main driver of demographic variation, in the majority of datasets, winter-related covariates were in the top models for both survival and recruitment, and 65% and 46% of these top models indicated that the effect was negative (decreased survival and decreased recruitment respectively). Although we might expect winter covariates to occur in top models 60% of the time by chance alone, a range of previous work highlights the importance of winter covariates. For example a synthesis of organismal responses to winter climate change reports that community composition, ecological interactions, and individual performance are affected by winter^39^. Other species-specific studies, including those focused on amphibians, indicate sensitivity to winter covariates^29,40,41^, and support our finding that populations are more sensitive to variation in winter conditions than conditions during the breeding and active seasons.

We hypothesized that we would see similar demographic responses to climate-related covariates across climatic zones and species, and that such uniformity might suggest that local responses to specific local conditions were being over-ridden. Contrary to our hypothesis, we identified a variety of responses both within and among amphibian species, suggesting that response to climate is highly context-dependent and that conservation planning will require a local approach despite the expected magnitude of climate change and potential for climate forcing. For example, there is a need to better determine how context-dependency can mediate the effect of climate on local populations and promote their resilience. Local contexts may include geographic, biotic and abiotic factors that differ in importance (e.g., climate forcing^42^; range position^43^; phenotypes^44^; geography^45^; habitat characteristics^35^). We expand on three examples from our analysis, but note that there are a number of interesting patterns that could be assessed further.

Three boreal toad populations in the Rocky Mountains occur in similar habitats, yet there was little uniformity in the survival model best supported by the data, and demographic response to covariates was often contrary to predictions. Models including the recurrence of warm events during winter (H5) had the most support, but affected survival as predicted (negatively) in only one population (at the highest elevation). High snowpack provides insulation during winter and lack of insulation can precipitate inappropriate rousing and waste of energy^40^. The criticality of this insulation, and thus the response in survival rate, likely depends on the elevation and physiogeography of local sites.

Results from great crested newts in France supported the importance of winter-related covariates (H5 and H4), but might also represent differences in demography related to the position of populations within the species’ range. Our newt populations are at the very southern edge of their range in contrast to newt studies in Britain^34^. The effect on survival of the number of warm days during hibernation (H5) had the most support in one population, but the direction of the effect, increased survival, was contrary to our prediction. Interestingly, results reported in Griffiths *et al*.^34^ – decreased survival with mild winters and heavy rain – support our H5. In our second newt dataset, survival decreased as winter severity increased (H4), as we predicted. Griffiths *et al*.^34^ provide evidence for the effect of climate factors at a regional level, but also asynchronies in subpopulation dynamics. Disparate results among our newt datasets echo these results.

There were also discrepancies compared to results published previously for Columbia spotted frogs. The best-supported model for one of two Columbia spotted frog populations in Montana suggested that survival increased as winter length (severity) increased, contrary to what we predicted, and contrary to an earlier analysis^46^. We attribute this to differences in characterization of winter covariates and possibly the inclusion of 5 additional years of data. This also highlights the challenge of using real field data to search for “cause” using correlational analyses.

Our analysis convincingly illustrates a pattern of local responses to similar climate-related stressors and fails to support an overarching effect of climate change on amphibian demographics. The variability we observed in demographic response, within and among species and populations, emphasizes the potential for species to adapt to local conditions and persist in changing environments, and is concomitant to the variability found among regions (i.e., different stressors in different regions, sensu^47^). These data provide population-level evidence to support a local, rather than a centralized, approach to conservation and illustrate the critical conservation need to maintain variability at every taxonomic level.

## Methods

### Species and sites

We used capture-mark-recapture data from pond-breeding amphibians (5 frogs, 3 toads and 2 newts), and one stream-breeding salamander (*Salamandra salamandra*) collected between 1965 and 2014. Eleven sites had 15–28 yr of data and 20 sites had 10–14 yr of data. Study sites were located in temperate regions; Mediterranean, montane or maritime climatic zones in Europe and North America (S1), and all animals were native to the locations where they were captured.

### Field methods

Animals were captured by hand or net; individual marks were applied to animals captured for the first time and existing marks (signifying recaptured animals) were recorded. Marking techniques varied by study and included passive integrated transponder tags, toe-clipping, visual implant elastomer, and identification based on photographs^48^. Data collection followed standard capture-mark-recapture study designs^49^ and all methods were carried out in accordance with relevant guidelines and regulations. In most studies, sites were visited at least three times during the breeding season each year and data conformed to Pollock’s robust design^49^. For datasets that included information for both sexes, we controlled for sex differences in survival and recruitment when modelling covariate effects.

### Analyses

We used the temporal-symmetry approach^50^ to estimate annual apparent survival, recruitment, and capture probabilities, and tested hypotheses about the influence of covariates on these parameters using a model selection approach. We assessed goodness-of-fit and accounted for potential over-dispersion using c-hat^49^. Apparent survival is defined as the probability of an adult individual surviving and not permanently emigrating from the study site between sampling occasions t and t + 1 and recruitment is defined as the ratio of new adults entering the focal population between year t and year t + 1 to the number of adults that were already present in the population (and exposed to capture) in sampling season t^50^. Recruitment encompasses two sources of new adults entering the focal population: 1) juveniles from the local population becoming adults and 2) immigration of adults from nearby populations. We assume that the majority of recruitment originated from local juvenile-to-adult transitions such that our measures of recruitment are representative of the local breeding process, although we acknowledge that this assumption may not hold for species with high dispersal rates^51^. Capture probability is the probability that a marked individual is available for detection (i.e., present at the site) and is detected during a sampling period. We used the robust design version of the Pradel model for all datasets except 7 and 8, where there was only a single visit per season, for those datasets we used the non-robust design version of the Pradel model^50^.

We developed a set of 9 *a priori* hypotheses (Table 1) to investigate the potential influence of environmental covariates with input from all the data providers. Hypotheses related environmental factors (water availability and temperature) to probabilities of survival (ϕ) and recruitment (f). Hypotheses were based on the reliance of our study species on standing water bodies and on our interpretation of the likely response (physiological or physical) of amphibians to extremes in temperatures. We developed five hypotheses for survival and four for recruitment.

In some cases, hypotheses were irrelevant for particular species and not assessed and in some of the recruitment hypotheses covariates were necessarily lagged (S1). For example, Hypothesis 4 (Table 1) is irrelevant for species inhabiting localities where snowpack does not occur, or for those species that hibernate aquatically. The covariates can reflect local variation as well as broad-scale change but hypotheses were developed considering local effects.

For each hypothesis a focal covariate was developed using daily precipitation, temperature, and snowpack data derived from local climate data (S6) specific for each study site. This strategy ensured that while we addressed consistent ecological hypotheses that might influence population dynamics, we allowed for the specific form of the influence to reflect local conditions. Because our interest was the mechanisms relating population fluctuations to relevant climate variables, this distinction, and focus on local conditions, is more appropriate than selecting a single consistent (large-scale) variable, and ensures that any deviation from expectation does not result from an inappropriate large-scale variable.

Models were implemented in Program MARK^52^ accessed through R (v. 3.1.0; R Development Core Team 2011), using the RMark interface^53^. Relative support for competing hypotheses was assessed via model selection^54^ performed in several steps, 1) select the best covariate structure for capture probability, keeping a full-time varying structure for survival and recruitment, 2) select the best covariate structure, regarding sex effects for survival and recruitment probabilities, and 3) assess the relative support for each hypothesis for each dataset. Models were selected using AIC corrected for small sample size (AIC_c_) and overdispersion (QAIC_c_) by a variance inflation factor (ĉ). For each dataset, ĉ was calculated (program RELEASE global test: TEST 2 + TEST 3; 51). A value of ĉ = 1 was assumed when the calculated ĉ was<1. QAIC_c_ weights were also used to assess the degree of support for each model. First, we assessed three hypotheses for capture probability: 1) *p* constant [p(.)]; 2) *p* as a linear function of capture occasion within each year; and 3) *p* as a quadratic function of capture occasion within each year. We tested for differences in detection probability between sexes when data were available. We selected the model with the most support for *p* and used that parameterization in all subsequent models. Next, we assessed the effect of sex on survival and recruitment for datasets with that information. If an effect of sex was supported, it was included in subsequent models. We then assessed the support for each hypotheses to quantifying the influence of each covariate on demographic parameters. Survival and recruitment are estimated simultaneously in Pradel models, thus, for each dataset, we assessed all possible combinations of 6 hypotheses for survival (H0-H5) and 5 hypotheses for recruitment (H0, H6-H9): 30 competing models (6 × 5 = 30 combinations). Hypothesis 4 was not relevant for 15 datasets (25 competing models (5 × 5 combinations). Parameter estimates from top models, and cumulative QAIC_c_ weights were used to quantify the degree of support for each hypothesis. We then compared the magnitude and the direction of influence of each covariate relative to *a priori* predictions.

We performed an analysis of deviance to quantify the amount of variation in survival and recruitment explained by the covariates^16^ (S6). This test relies on an F-statistic based on the deviances, and the corresponding degrees of freedom, of models assuming full-time variation (F_t_), constancy (F_0_) and covariate-driven variation (F_H_, our nine covariates) for the parameter of interest (e.g., survival), and assuming the best supported parameterization for the other model parameters (i.e., capture probability and recruitment). Using ratios of deviances from these models, we calculated the proportion of variation (r²) explained by each covariate (r² = Dev(F_0_) − Dev(F_H_)/Dev(F_0_) − Dev(F_t_)). We assessed the amount of variation explained by grouping data sets by taxonomy; life history (i.e., hibernation site – aquatic vs terrestrial); and by environment (maritime, Mediterranean, or montane) (S6A, B).

We ran full-time varying models for survival (Φ_t_) and recruitment (f_t_) to provide annual estimates of these parameters, for each site. We plotted annual estimates and predicted annual values from the best supported covariate model (S3). We subsequently derived inter-annual values of population growth λ_t_, as λ_t_ = Φ_t_ + f_t_ for each and plotted annual estimates to illustrate the trajectory for each population (S7).

### Data Availability

Derived data are available in the online appendices and at the John Wesley Powell Center for Synthesis and Analysis (https://powellcenter.usgs.gov/data-resources). Raw data are available from the authors of each respective dataset.

## Electronic supplementary material

Supplementary online material
