# Supplementary material for: Heterogeneous responses of temperate-zone amphibian populations to climate change complicates conservation planning
Source: Sci Rep. 2017 Dec 6;7:17102. doi: 10.1038/s41598-017-17105-7 (PMC5719039; doi:10.1038/s41598-017-17105-7)

SUPPLEMENTARY MATERIAL FOR:

Heterogeneous responses of temperate-zone amphibian populations to climate change complicates conservation planning[1]

Authors: E. Muths[1*], T. Chambert[2,3], B.R. Schmidt[4,5], D.A.W. Miller[2], B.R. Hossack[6], P. Joly[7], O. Grolet[7], D.M. Green[8], D.S. Pilliod[9], M. Cheylan[10], R.N. Fisher[11], R.M. McCaffery[12], M.J. Adams[13], W. Palen[14] , J. W. Arntzen[15], J. Garwood[16], G. Fellers[17], J-M. Thirion[18] A. Besnard[10], E.H. Campbell Grant[19]

Affiliations:
[1]U.S. Geological Survey, Fort Collins Science Center Fort Collins, CO 80526, USA
[2]Pennsylvania State University, Department of Ecosystem Science and Management, University Park, PA 16802, USA
[3]U.S. Geological Survey, Patuxent Wildlife Research Center, Laurel, MD 20708, USA
[4]Department of Evolutionary Biology and Environmental Studies, University of Zurich, 8057 Zurich, Switzerland; benedikt.schmidt@ieu.uzh.ch.
[5]KARCH, 2000 Neuchâtel, Switzerland
[6]U.S. Geological Survey, Aldo Leopold Wilderness Research Institute, Missoula, MT 59801
[7]Université Lyon 1, UMR 5023 - LEHNA, Laboratoire d'Ecologie des Hydrosystèmes Naturels et Anthropisés, 69100 Villeurbanne, France; pierre.joly@univ-lyon1.fr; +33 04 72 35 86; Odile.Grolet@univ-lyon1.fr
[8]Redpath Museum, McGill University, 859 Sherbrooke St. W. Montreal, Quebec H3A 2K6, Canada. david.m.green@mcgill.ca; 514-3998-4088.
[9]U.S. Geological Survey, Forest and Rangeland Ecosystem Science Center, 970 Lusk St., Boise, ID 83706, USA. dpilliod@usgs.gov; 208-426-5202
Survey Snake River Field Station, Boise, ID
[10]CNRS, PSL Research University, EPHE, UM, SupAgro, IRD, INRA, UMR 5175 CEFE, F-34293 Montpellier, France marc.cheylan@cefe.cnrs.fr; +33 4 67 61 32 61;
aurelien.besnard@cefe.cnrs.fr ; +33 (0)4 67 61 32 94
[11]U.S. Geological Survey, Western Ecological Research Center, San Diego Field Station, 4165 Spruance Road, San Diego, CA 92101, USA.
[12]University of Montana, Missoula, MT; current affiliation: U.S. Geological Survey, Olympic field station, Forest and Rangeland Ecosystem Science Center, Port Angeles, WA 98362, USA
[13]U.S. Geological Survey, Forest and Rangeland Ecosystem Science Center, Corvallis, OR 97331, USA
[14] Simon Fraser University, Department of Biological Sciences, 8888 University Drive
Burnaby, British Columbia CANADA V5A 1S6; wpalen@sfu.ca; 778-782-4063.
[15]Naturalis Biodiversity Center, 6.4.16 Sylvius Bldg, 2333 CR Leiden, The Netherlands; pim.arntzen@naturalis.nl; +31 (0)71-52 71 921.
[16]California Department of Fish and Wildlife, 5341 Ericson Way, Arcata, CA 95521, USA; justin.garwood@wildlife.ca.gov
[17]U.S. Geological Survey, Western Ecological Research Center, Point Reyes Field Station, Point Reyes National Seashore, Point Reyes, CA 94956, USA; gary_fellers@usgs.gov; +1-415-464-5185
[18]Association Objectifs Biodiversités (OBIOS), 12 rue du docteur Gilbert, 17250 Pont l'Abbé d'Arnoult, France. thirion.jean-marc@sfr.fr; +33 5 46 97 12 38.
[19]U.S. Geological Survey, Patuxent Wildlife Research Center, SO Conte Anadromous Fish Laboratory, Turners Falls, MA 01342, USA; ehgrant@usgs.gov; +1-413-863-3823.
*Correspondence to: e-mail: muthse@usgs.gov; +1 (970) 226-9474

[1]After first 5 authors, authors are listed based on the length of the contributed data set, if sets the same length, authors listed alphabetically (except for the final author).

Supplementary Information 1: Information about data sets (location, zone, elevation, length of data set, species and species details

data set	Country	State	Department or County	Zone: Montane=MON; Mediterranean=MED; Maritime=MAR*	Elevation (m)	# of yrs of data	Snowpack relevant (Y/N)	Species	species abbreviation	Age (yrs) at maturity (M)	Age (yrs) at maturity (F)	Age (yrs) at metamorphosis	Approximate date of egg deposition	Approximate end of breeding season	Approximate date of metamorphosis	Hibernation: Terrestrial (T) or Aquatic (A)
1	France		Mayenne	MAR	122	12	N	*Triturus marmoratus*	TRMA	4	4	0	25-Apr	25-May	25-Aug	T
2	France		Charente	MAR	137	10	N	*Bombina variegata*	BOVA	3	3	0	30-Apr	30-May	15-Jun	T
3	France		Gard	MED	140	14	N	*Triturus cristatus*	TRCR	2	2	0	1-Mar	31-Mar	15-Jul	T
4	France		Charente	MAR	156	10	N	*Bombina variegata*	BOVA	3	3	0	30-Apr	30-May	15-Jun	T
5	Canada	Ontario		MAR	174	17	N	*Anaxyrus fowleri*	ANFO	2	2	0	2-Jun	2-Jul	22-Jul	T
6	France		Ain	MAR	305	22	N	*Triturus cristatus*	TRCR	2	3	0	15-Apr	15-May	31-Jul	T
7	Germany	Nordrhein-Westfalen		MAR	410	18	N	*Salamandra salamandra*	SASA	4	4	1	1-Apr	1-May	1-Jun	T
8	Germany	Nordrhein-Westfalen		MAR	450	21	N	*Salamandra salamandra*	SASA	4	4	1	1-Apr	1-May	1-Jun	T
9	USA	California	San Bernadino	MED	859	14	N	*Rana muscosa*	RAMU	4	4	1	15-May	14-Jun	15-Aug	A
10	USA	Montana	Missoula	MON	1230	11	Y	*Anaxyrus boreas*	ANBO	3	4	0	19-May	18-Jun	17-Jul	T
11	USA	Washington	Clallum	MON	1293	12	N	*Rana cascadae*	RACA	3	3	0	23-Jun	23-Jul	7-Sep	A
12	USA	California	Los Angeles	MED	1547	14	N	*Rana muscosa*	RAMU	4	4	1	15-May	14-Jun	15-Aug	A
13	USA	California	Los Angeles	MED	1628	13	N	*Rana muscosa*	RAMU	4	4	1	15-May	14-Jun	15-Aug	A
14	USA	California	Los Angeles	MED	1643	14	N	*Rana muscosa*	RAMU	4	4	1	15-May	14-Jun	15-Aug	A
15	USA	California	Los Angeles	MED	1722	15	N	*Rana muscosa*	RAMU	4	4	1	15-May	14-Jun	15-Aug	A
16	USA	Idaho	Owyhee	MON	1774	16	N	*Rana luteiventris*	RALU	2	2	0	28-Apr	28-May	25-Jul	A
17	USA	California	Riverside	MED	1775	12	N	*Rana muscosa*	RAMU	4	4	1	15-May	14-Jun	15-Aug	A
18	USA	California	Riverside	MED	1804	13	N	*Rana muscosa*	RAMU	4	4	1	15-May	14-Jun	15-Aug	A
19	USA	California	Los Angeles	MED	1932	14	N	*Rana muscosa*	RAMU	4	4	1	15-May	14-Jun	15-Aug	A
20	USA	Montana	Bitterroot	MON	1995	13	N	*Rana luteiventris*	RALU	4	4	0	5-Jun	5-Jul	25-Aug	A

21	USA	Wyoming	Teton	MON	2085	12	Y	*Anaxyrus boreas*	ANBO	3	5	0	21-May	20-Jun	20-Jul	T
22	USA	California	Trinity	MON	2100	12	Y	*Rana cascadae*	RACA	4	5	0	7-Jun	1-Jul	15-Sep	A
23	USA	Montana	Bitterroot	MON	2200	14	N	*Rana luteiventris*	RALU	4	4	0	19-Jun	19-Jul	7-Sep	A
24	USA	California	Mariposa	MON	2200	11	N	*Rana Sierrae*	RASI	6	6	2	5-Jun	5-Jul	15-Aug	A
25	USA	Idaho	Lemhi	MON	2478	10	N	*Rana luteiventris*	RALU	3	3	0	20-Jun	20-Jul	4-Sep	A
26	USA	Colorado	Larimer	MON	2803	28	Y	*Pseudacris maculata*	PSMA	3	3	0	30-Apr	30-May	20-Jul	T
27	USA	Colorado	Larimer	MON	2810	23	Y	*Anaxyrus boreas*	ANBO	4	4	0	1-Jun	1-Jul	25-Jul	T
28	USA	Colorado	Larimer	MON	2946	13	Y	*Anaxyrus boreas*	ANBO	4	4	0	15-Jun	15-Jul	25-Aug	T
29	USA	Colorado	Larimer	MON	2969	28	Y	*Pseudacris maculata*	PSMA	3	3	0	7-Jun	7-Jul	30-Jul	T
30	USA	Colorado	Chaffee	MON	3138	15	Y	*Anaxyrus boreas*	ANBO	4	4	0	25-May	24-Jun	1-Sep	T
31	USA	Colorado	Larimer	MON	3266	23	Y	*Anaxyrus boreas*	ANBO	4	4	0	15-Jun	15-Jul	25-Aug	T

*NOTE: specific locations, e.g. geographic coordinates, have been redactd to protect location of endangered or threatened species.

Supplementary Information 2: Capture probability; graphs of survival and recruitment probabilities

PROBABILITY OF DETECTION

species	dataset	average	min	max	average by taxa
TRMA	1	0.22	0.174	0.284	newts / salamanders
TRCR	3	0.164	0.107	0.314	0.4304
TRCR	6	0.564	0.477	0.65	
SASA	7	0.58	0.406	0.743	
SASA	8	0.624	0.461	0.697	
RAMU	9	0.096	0.096	0.096	ranid frogs / tree frogs
RACA	11	0.309	0.169	0.427	0.159294118
RAMU	12	0.122	0.122	0.122	
RAMU	13	0.1	0.035	0.189	
RAMU	14	0.173	0.089	0.287	
RAMU	15	0.157	0.086	0.242	
RALU	16	0.06	0.008	0.201	
RAMU	17	0.165	0.093	0.25	
RAMU	18	0.143	0.075	0.225	
RAMU	19	0.286	0.048	0.473	
RALU	20	0.181	0.15	0.213	
RACA	22	0.354	0.162	0.64	
RALU	23	0.265	0.171	0.371	
RASI	24	0.129	0.078	0.172	
RALU	25	0.041	0.008	0.099	
PSMA	26	0.087	0.018	0.18	
PSMA	29	0.04	0.007	0.101	
BOVA	2	0.209	0.09	0.303	bufonids
BOVA	4	0.249	0.072	0.462	0.170444444
ANFO	5	0.235	0.121	0.377	
ANBO	10	0.255	0.09	0.541	
ANBO	21	0.058	0.004	0.178	
ANBO	27	0.037	0	0.151	
ANBO	28	0.203	0.006	0.371	
ANBO	30	0.254	0.227	0.281	
ANBO	31	0.034	0.001	0.095	

SURVIVAL MARITIME

Graphs illustrate the estimated effect of the covariate that received the most support from the data (i.e., the top model). The X-axis is in the units of that covariate (i.e., the variable we used to assess the hypotheses [Table 2, covariate and formulaton]. Dots represent the yearly estimates from the t model (phi(t), f(t), p(.)). The line represents the predicted values from the model best supported by the data (e.g., Phi(H5[t]) = logit-1 (beta0 + beta(H5)[t])). Note: the scale is NOT the same on all graphs. Species code: ANFO - *Anaxyrus fowleri*, BOVA - *Bombina variegata*, SASA - *Salamandra salamandra*, TRCR - *Triturus cristatus*, TRMA - *T. marmoratus*.

TRMA $r^2 = 0.68$ 1

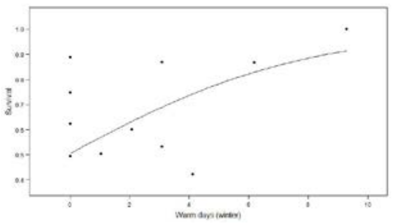

BOVA $r^2 = 0.20$ 2

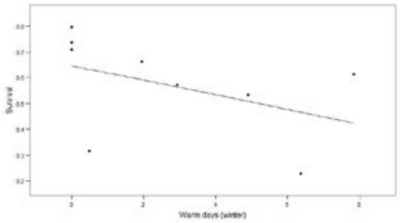

BOVA $r^2 = 0.14$ 4

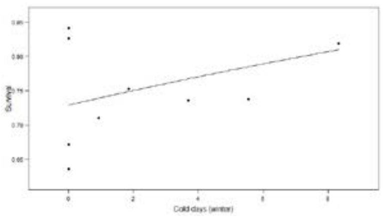

ANFO $r^2 = 0.21$ 5

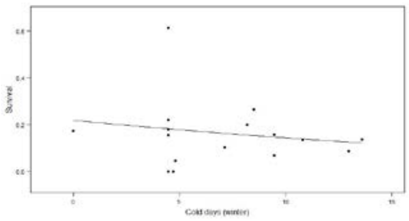

TRCR $r^2 = 0.38$ 6

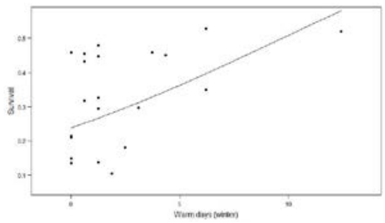

SASA $r^2 = 0.44$ 7

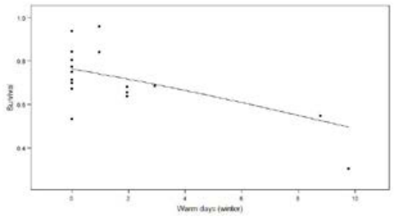

SASA $r^2 = 0.14$ 8

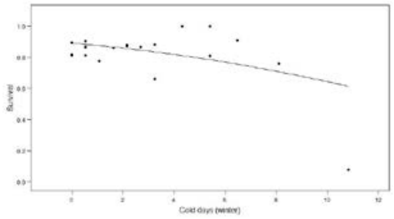

SURVIVAL MEDITERRANEAN

Graphs illustrate the estimated effect of the covariate that received the most support from the data (i.e., the top model). The X-axis is in the units of that covariate (i.e., the variable we used to assess the hypotheses [Table 2, covariate and formulaton]. Dots represent the yearly estimates from the t model (phi(t), f(t), p(.)). The line represents the predicted values from the model best supported by the data (e.g., Phi(H5[t]) = logit-1 (beta0 + beta(H5)[t])). Note: the scale is NOT the same on all graphs. Species code: Species code: TRCR - *Triturus cristatus*, RAMU - *Rana muscosa*.

TRCR $r^2 = 0.10$ 3

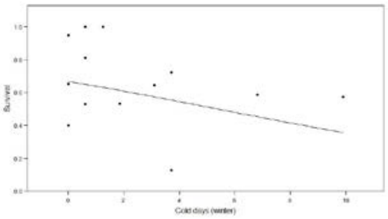

RAMU $r^2 = 0.32$ 15

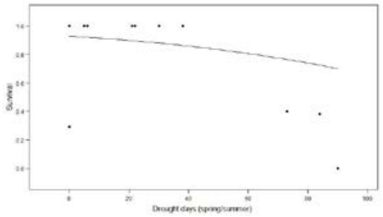

RAMU $r^2 = 0.28$ 9

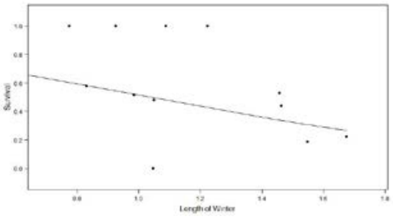

RAMU Dark Canyo 17

survival constant

RAMU $r^2 = 0.39$ 12

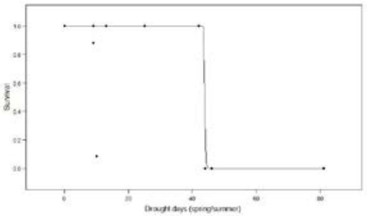

RAMU $r^2 = 0.29$ 18

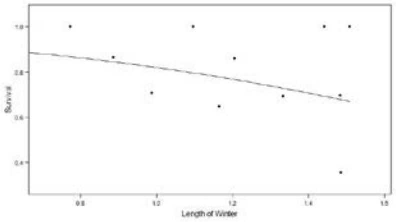

RAMU $r^2 = 0.69$ 13

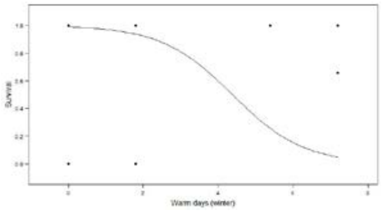

RAMU $r^2 = 0.28$ 19

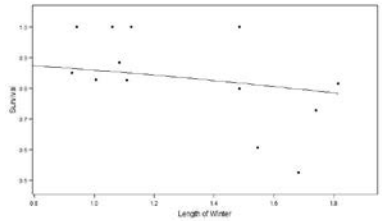

RAMU $r^2 = 0.30$ 14

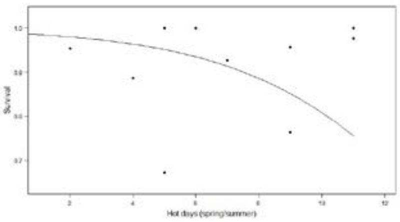

SURVIAL MONTANE

Graphs illustrate the estimated effect of the covariate that received the most support from the data (i.e., the top model). The X-axis is in the units of that covariate (i.e., the variable we used to assess the hypotheses [Table 2, covariate and formulaton]. Dots represent the yearly estimates from the t model (phi(t), f(t), p(.)). The line represents the predicted values from the model best supported by the data (e.g., Phi(H5[t]) = logit-1 (beta0 + beta(H5)[t])). Note: the scale is NOT the same on all graphs. Species code: ANBO - *Anaxyrus boreas*, PSMA - *Pseudacris maculata*, RACA - *Rana cascadae*, RALU - *R. lutieventris*, RASI - *R. sierrae*.

ANBO $r^2 = 0.46$ 10

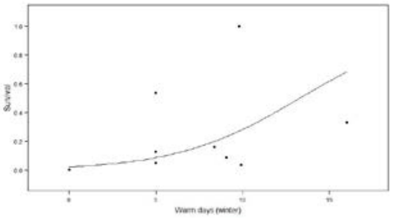

RACA $r^2 = 0.27$ 22

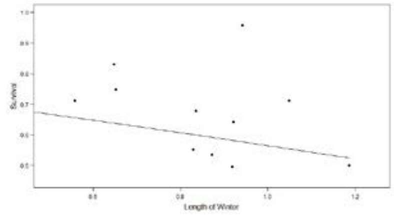

ANBO $r^2 = 0.54$ 27

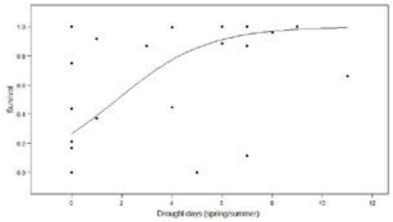

RACA $r^2 = 0.19$ 11

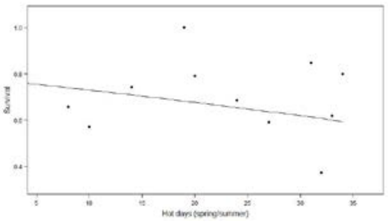

RALU $r^2 = 0.40$ 23

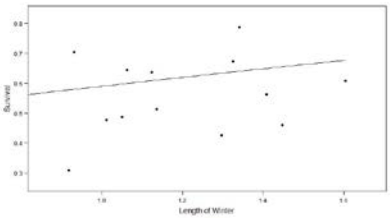

ANBO $r^2 = 0.21$ 28

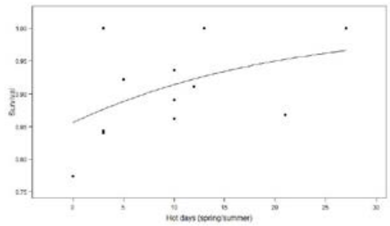

RALU $r^2 = 0.55$ 16

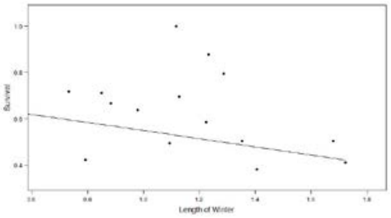

RASI $r^2 = 0.54$ 24

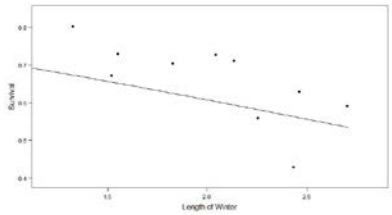

PSMA $r^2 = 0.10$ 29

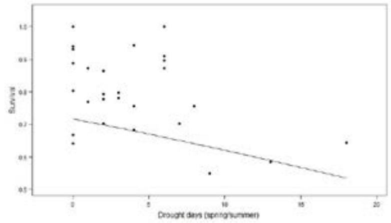

RALU $r^2 = 0.07$ 20

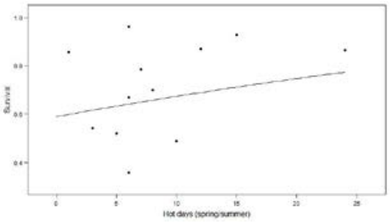

RALU $r^2 = 0.32$ 25

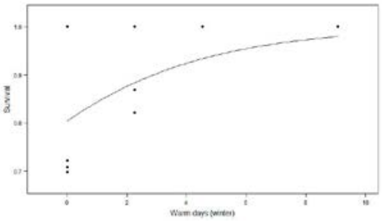

ANBO $r^2 = 0.09$ 30

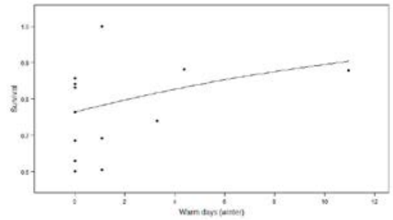

ANBO $r^2 = 0.48$ 21

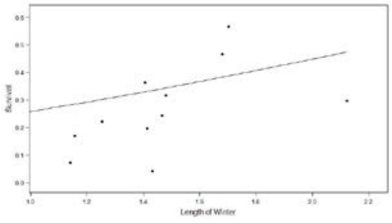

PSMA $r^2 = 0.17$ 26

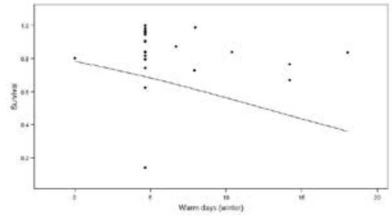

ANBO $r^2 = 0.48$ 31

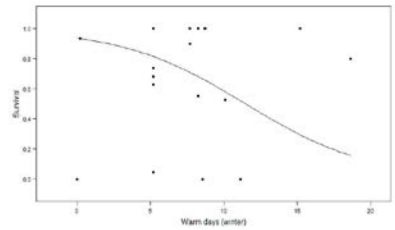

RECRUITMENT MARITIME

Graphs illustrate the estimated effect of the covariate that received the most support from the data (i.e., the top model). The X-axis is in the units of that covariate (i.e., the variable we used to assess the hypotheses [Table 2, covariate and formulaton]. Dots represent the yearly estimates from the t model (phi(t), f(t), p(.)). The line represents the predicted values from the model best supported by the data (e.g., Phi(H5[t]) = exp (beta0 + beta(H5)[t])). Note: the scale is NOT the same on all graphs. Species code: ANFO - *Anaxyrus fowleri*, BOVA - *Bombina variegata*, SASA - *Salamandra salamandra*, TRCR - *Triturus cristatus*, TRMA - *T. marmoratus*.

TRMA $r^2 = 0.41$ 1

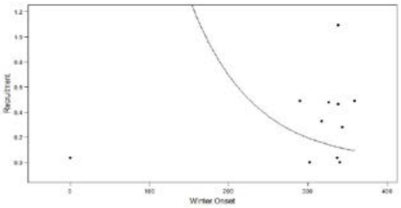

BOVA Plaine 2

recruitment constant

BOVA $r^2 = 0.35$ 4

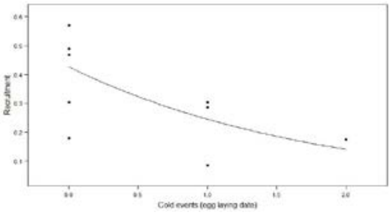

ANFO $r^2 = 0.03$ 5

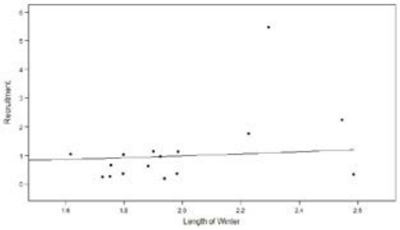

TRCR $r^2 = 0.23$ 6

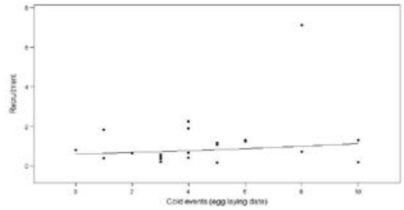

SASA $r^2 = 0.19$ 7

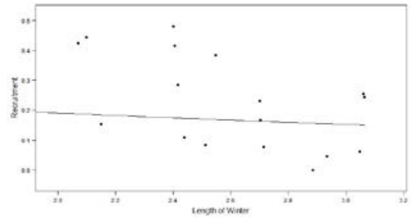

SASA $r^2 = 0.05$ 8

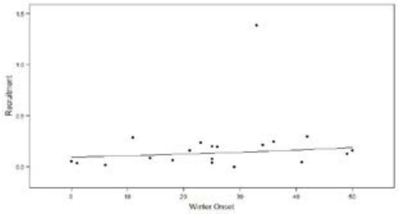

RECRUITMENT MEDITERRANEAN

Graphs illustrate the estimated effect of the covariate that received the most support from the data (i.e., the top model). The X-axis is in the units of that covariate (i.e., the variable we used to assess the hypotheses [Table 2, covariate and formulaton]. Dots represent the yearly estimates from the t model (phi(t), f(t), p(.)). The line represents the predicted values from the model best supported by the data (e.g., Phi(H5[t]) = exp (beta0 + beta(H5)[t])). Note: the scale is NOT the same on all graphs. Species code: Species code: TRCR - *Triturus cristatus*, RAMU - *Rana muscosa*.

TRCR $r^2 = 0.40$ 3

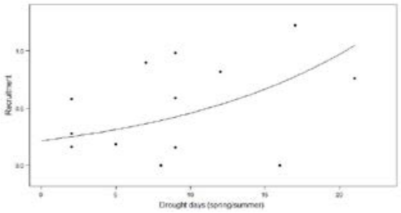

RAMU $r^2 = 0.14$ 9

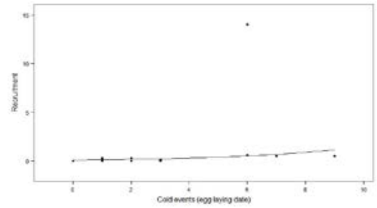

RAMU $r^2 = 0.69$ 12

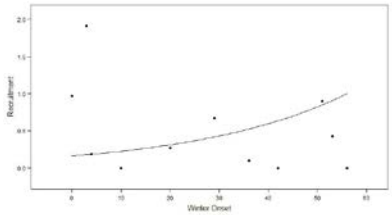

RAMU $r^2 = 0.29$ 13

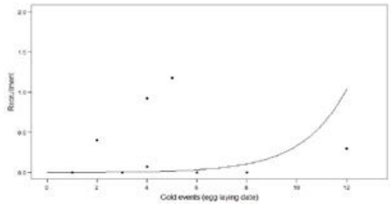

RAMU $r^2 = 0.14$ 14

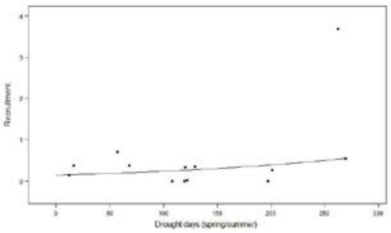

RAMU $r^2 = 0.50$ 15

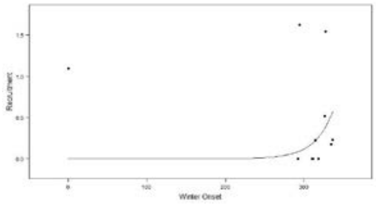

RAMU $r^2 = 0.53$ 17

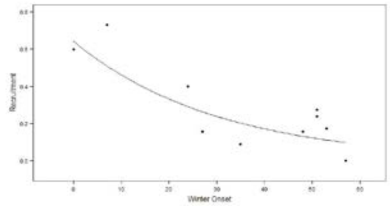

RAMU $r^2 = 0.44$ 18

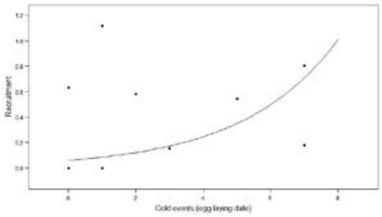

RAMU $r^2 = 0.06$ 19

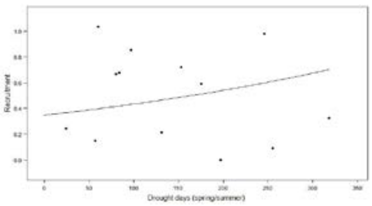

RECRUITMENT MONTANE

Graphs illustrate the estimated effect of the covariate that received the most support from the data (i.e., the top model). The X-axis is in the units of that covariate (i.e., the variable we used to assess the hypotheses [Table 2, covariate and formulaton]. Dots represent the yearly estimates from the t model (phi(t), f(t), p(.)). The line represents the predicted values from the model best supported by the data (e.g., Phi(H5[t]) = exp (beta0 + beta(H5)[t])). Note: the scale is NOT the same on all graphs. Species code: ANBO - *Anaxyrus boreas*, PSMA - *Pseudacris maculata*, RACA - *Rana cascadae*, RALU - *R. lutieventris*, RASI - *R. sierrae*.

ANBO $r^2 = 0.33$ 10

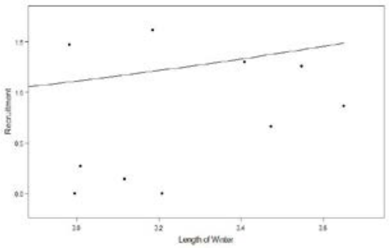

RACA $r^2 = 0.36$ 22

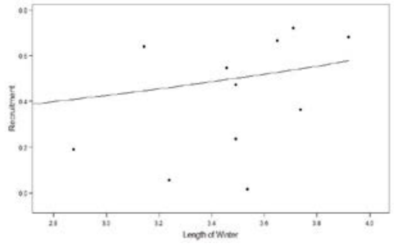

ANBO $r^2 = 0.46$ 27

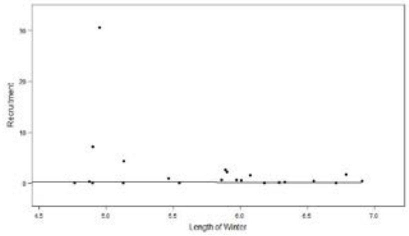

RACA $r^2 = 0.06$ 11

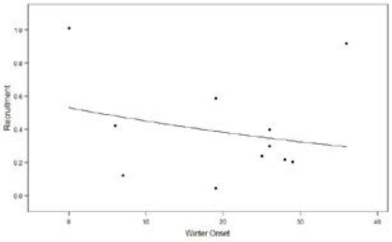

RALU $r^2 = 0.22$ 23

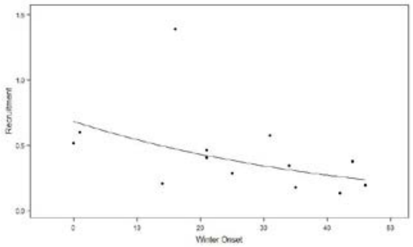

ANBO $r^2 = 0.37$ 28

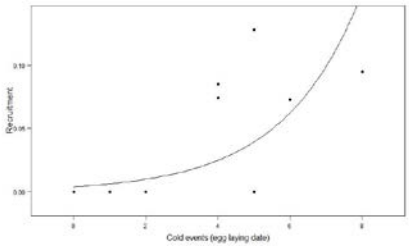

RALU $r^2 = 0.16$ 16

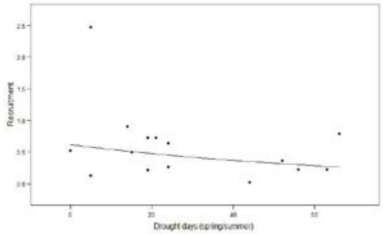

RASI $r^2 = 0.13$ 24

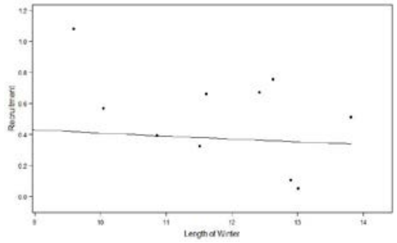

PSMA $r^2 = 0.01$ 29

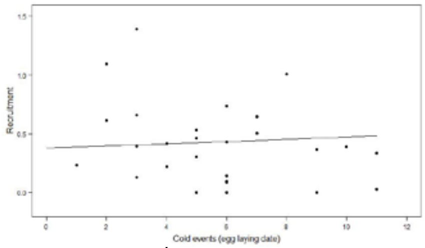

RALU $r^2 = 0.15$ 20

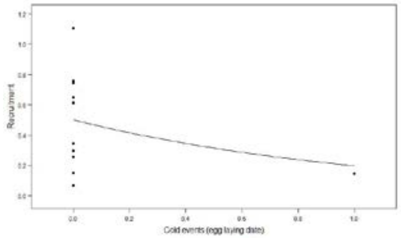

RALU $r^2 = 0.39$ 25

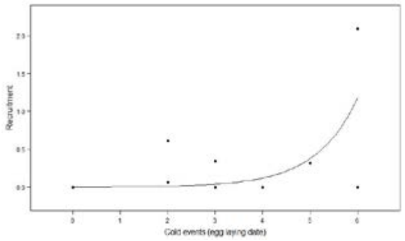

ANBO $r^2 = 0.28$ 30

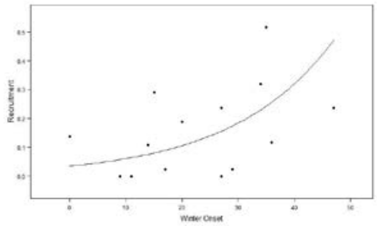

ANBO $r^2 = 0.29$ 21

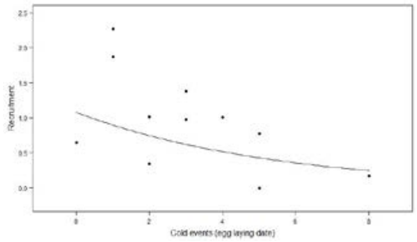

PSMA Matthews 26

recruitment constant

ANBO $r^2 = 0.08$ 31

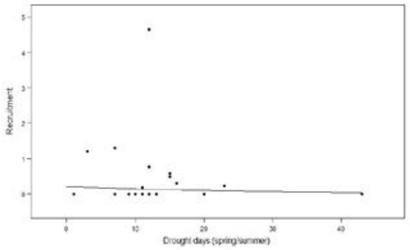

Supplementary Information 3: Model selection results

H1 – H6: hypotheses about survival; H7-H9: hypotheses about recruitment. Models where parameters are constant are indictated by ".". Φ = probability of survival; *f* = probability of recruitment; *p* = probability of detection, k = number of parameters in the model; AIC_c = Akaike's information criterion corrected for small sample sizes; w_i = model AIC_c weight. Modesl that received no weight are not shown. See Table 1 for details of each data set.

Dataset	Species	Φ	*f*	*p*	*k*	AIC_c	ΔAIC_c	w_i	Deviance
1	*Triturus marmoratus*	H5 + sex	H9	sex + date + date^2	9	3656.71	0	0.93	3638.42
		H1 + sex	H9	sex + date + date^2	9	3661.99	5.28	0.07	3643.7
2	*Bombina variegata*	H5	sex	sex + date + date^2	8	6375.4	0	0.23	4724.47
		H5	H6 + sex	sex + date + date^2	9	6375.68	0.28	0.2	4722.72
		H5	H9 + sex	sex + date + date^2	9	6375.71	0.31	0.2	4722.75
		H5	H7 + sex	sex + date + date^2	9	6375.73	0.33	0.2	4722.77
		H5	H8 + sex	sex + date + date^2	9	6377.25	1.85	0.09	4724.29
		H1	H6 + sex	sex + date + date^2	9	6382.04	6.63	0.01	4729.07
		H1	H9 + sex	sex + date + date^2	9	6382.48	7.07	0.01	4729.51
		.	H9 + sex	sex + date + date^2	8	6382.69	7.29	0.01	4731.76
3	*Triturus cristatus*	H4	H6	sex + date + date^2	8	13552.5	0	1	13536.43
4	*Bombina variegata*	H4	H8	sex + date + date^2	8	9080.01	0	0.17	9063.93
		.	H8	sex + date + date^2	7	9080.26	0.25	0.15	9066.2
		H5	H8	sex + date + date^2	8	9080.55	0.54	0.13	9064.47
		H2	H8	sex + date + date^2	8	9081.49	1.48	0.08	9065.42
		H3	H8	sex + date + date^2	8	9081.96	1.95	0.06	9065.89
		H4	H6	sex + date + date^2	8	9082.08	2.07	0.06	9066.01
		H1	H8	sex + date + date^2	8	9082.17	2.16	0.06	9066.1
		H4	H7	sex + date + date^2	8	9082.83	2.82	0.04	9066.76
		.	H7	sex + date + date^2	7	9083.14	3.13	0.04	9069.08

		.	H6	sex + date + date^2	7	9083.2	3.19	0.03	9069.14
		H2	H6	sex + date + date^2	8	9083.4	3.39	0.03	9067.32
		H5	H6	sex + date + date^2	8	9084.01	4	0.02	9067.93
		H3	H7	sex + date + date^2	8	9084.15	4.14	0.02	9068.07
		H1	H6	sex + date + date^2	8	9084.3	4.29	0.02	9068.22
		H1	H7	sex + date + date^2	8	9084.37	4.36	0.02	9068.29
		H5	H7	sex + date + date^2	8	9084.49	4.48	0.02	9068.41
		H2	H7	sex + date + date^2	8	9084.64	4.63	0.02	9068.57
		H3	H6	sex + date + date^2	8	9084.97	4.96	0.01	9068.89
5	*Anaxyrus fowleri*	H4	H7	date	6	11808.03	0	0.82	11795.98
		H3	H7	date	6	11812.78	4.75	0.08	11800.73
		.	H7	date	5	11813.75	5.72	0.05	11803.71
		H2	H7	date	6	11815.19	7.16	0.02	11803.14
		H1	H7	date	6	11815.48	7.45	0.02	11803.42
		H5	H7	date	6	11815.73	7.7	0.02	11803.68
6	*Triturus cristatus*	H5	H8 + sex	sex + date	8	21847.56	0	1	21831.53
7	*Salamandra salamandra*	H5	date	H7	6	1913.87	0	0.41	428.75
		H5	date	H9	6	1915.78	1.91	0.16	430.66
		H5	date	.	5	1916.32	2.45	0.12	433.26
		H3	date	H7	6	1917.13	3.26	0.08	432.01
		H5	date	H8	6	1918.01	4.13	0.05	432.89
		H5	date	H6	6	1918.21	4.34	0.05	433.09
		.	date	H7	5	1919.36	5.49	0.03	436.3
		H3	date	H9	6	1919.61	5.73	0.02	434.48
		H3	date	.	5	1920.05	6.18	0.02	436.98
		H1	date	H7	6	1920.42	6.54	0.02	435.29
		H2	date	H7	6	1920.92	7.05	0.01	435.8
		H4	date	H7	6	1921.13	7.25	0.01	436.01
		H3	date	H8	6	1921.73	7.86	0.01	436.61
		H3	date	H6	6	1921.89	8.02	0.01	436.77

8	*Salamandra salamandra*	H4	date + date^2	H9	7	5480.9	0	0.35	1713.68
		H4	date + date^2	H7	7	5482.09	1.19	0.19	1714.87
		H4	date + date^2	H8	7	5482.19	1.29	0.18	1714.97
		H4	date + date^2	.	6	5482.52	1.62	0.15	1717.32
		H4	date + date^2	H6	7	5483.01	2.11	0.12	1715.79
9	*Rana muscosa*	H3	H8	.	5	659.35	0	0.53	320.22
		H3	H8	.	5	662.47	3.12	0.11	323.34
		H3	.	.	4	662.87	3.51	0.09	326
		H3	H6	.	5	663.49	4.14	0.07	324.36
		H2	H8	.	5	663.82	4.47	0.06	324.69
		.	H8	.	4	664.69	5.34	0.04	327.82
		H3	H7	.	5	664.79	5.43	0.04	325.65
		H5	H8	.	5	665.44	6.09	0.03	326.31
		H1	H8	.	5	666.63	7.28	0.01	327.5
10	*Anaxyrus boreas*	H5 + sex	H7	sex + date + date^2	9	5251.34	0	0.99	5233.18
		H2 + sex	H7	sex + date + date^2	9	5260.2	8.85	0.01	5242.03
11	*Rana cascadae*	H2 + sex	H9 + sex	sex + date + date^2	10	10292.66	0	0.64	10272.56
		H2 + sex	H6 + sex	sex + date + date^2	10	10294.87	2.2	0.21	10274.76
		H2 + sex	sex	sex + date + date^2	9	10297.4	4.74	0.06	10279.32
		H2 + sex	H8 + sex	sex + date + date^2	10	10298.17	5.51	0.04	10278.06
		H2 + sex	H7 + sex	sex + date + date^2	10	10298.87	6.2	0.03	10278.76
		H5 + sex	H9 + sex	sex + date + date^2	10	10300.38	7.71	0.01	10280.27
12	*Rana muscosa*	H1	H9	.	5	511.98	0	0.3	218.02
		H1	H6	.	5	512.52	0.54	0.23	218.57
		H3	.	.	4	513.8	1.82	0.12	222.14
		H3	H9	.	5	514.39	2.41	0.09	220.43
		H3	H6	.	5	514.41	2.43	0.09	220.45
		H3	H8	.	5	514.82	2.84	0.07	220.87
		H1	H7	.	5	515.13	3.15	0.06	221.17
		H3	H7	.	5	515.6	3.62	0.05	221.65

13	*Rana muscosa*	H5	H8	date	6	401.57	0	0.32	145.14
		H5	H6	date	6	401.97	0.4	0.26	145.54
		H5	H7	date	6	403.11	1.55	0.15	146.69
		H5	.	date	5	403.24	1.67	0.14	149.21
		H5	H9	date	6	403.29	1.73	0.13	146.87
14	*Rana muscosa*	H2 + sex	H6}	sex + date	8	2518.55	0	0.63	1547
		H1 + sex	H6	sex + date	8	2522.59	4.04	0.08	1551.03
		H2 + sex	H9	sex + date	8	2523.71	5.16	0.05	1552.16
		sex	H9	sex + date	7	2524.78	6.23	0.03	1555.31
		H2 + sex	H7	sex + date	8	2524.9	6.35	0.03	1553.35
		H5 + sex	H6	sex + date	8	2524.97	6.42	0.03	1553.41
		sex	H6	sex + date	7	2525.27	6.72	0.02	1555.8
		H5 + sex	H9	sex + date	8	2525.43	6.88	0.02	1553.88
		sex	H7	sex + date	7	2525.5	6.95	0.02	1556.03
		H3 + sex	H6	sex + date	8	2526.23	7.68	0.01	1554.68
		H5 + sex	H7	sex + date	8	2526.25	7.7	0.01	1554.69
		H3 + sex	H9	sex + date	8	2526.59	8.04	0.01	1555.04
		H1 + sex	H9	sex + date	8	2526.73	8.18	0.01	1555.18
		H2 + sex	.	sex + date	7	2526.79	8.24	0.01	1557.32
		H2 + sex	H8	sex + date	8	2527.22	8.67	0.01	1555.66
		H1 + sex	H7	sex + date	8	2527.36	8.81	0.01	1555.8
		H3 + sex	H7	sex + date	8	2527.42	8.87	0.01	1555.87
15	*Rana muscosa*	H5	H8	date + date^2	7	1036.4	0	1	469.4
16	*Rana luteiventris*	H3 + sex	H6 + sex	sex + date + date^2	13	38623.35	0	1	38597.28
17	*Rana muscosa*	.	H9	date	5	1719.13	0	0.18	840.39
		.	H7	date	5	1719.67	0.54	0.14	840.93
		H3	H7	date	6	1720.2	1.07	0.11	839.38
		H3	H9	date	6	1720.47	1.34	0.09	839.65

		H1	H9	date	6	1720.52	1.39	0.09	839.7
		H2	H9	date	6	1720.56	1.43	0.09	839.73
		H2	H7	date	6	1720.66	1.52	0.08	839.83
		H1	H7	date	6	1720.66	1.53	0.08	839.83
		H5	H9	date	6	1721.1	1.97	0.07	840.28
		H5	H7	date	6	1721.7	2.57	0.05	840.88
18	*Rana muscosa*	H3	H8	date	6	676.21	0	0.43	371.57
		H2	H8	date	6	677.18	0.97	0.27	372.54
		.	H8	date	5	679.35	3.14	0.09	376.96
		H5	H8	date	6	681.19	4.98	0.04	376.55
		H1	H8	date	6	681.54	5.33	0.03	376.9
		H2	H7	date	6	682.89	6.68	0.02	378.25
		H2	.	date	5	682.89	6.68	0.02	380.5
		H1	H7	date	6	682.9	6.69	0.02	378.26
		H3	H7	date	6	682.95	6.75	0.01	378.31
		H3	.	date	5	683.04	6.83	0.01	380.65
		.	H7	date	5	683.17	6.97	0.01	380.78
		H5	H7	date	6	683.69	7.48	0.01	379.05
		H3	H9	date	6	683.88	7.67	0.01	379.24
		H2	H9	date	6	684.02	7.81	0.01	379.38
		H3	H6	date	6	685.08	8.87	0.01	380.44
		H2	H6	date	6	685.13	8.92	0.01	380.49
19	*Rana muscosa*	H3 + sex	H6	sex + date + date^2	9	3671.93	0	0.25	1841.58
		H3 + sex	.	sex + date + date^2	8	3672.51	0.58	0.19	1844.2
		H3 + sex	H8	sex + date + date^2	9	3672.96	1.03	0.15	1842.61
		H3 + sex	H7	sex + date + date^2	9	3673.82	1.89	0.1	1843.47
		H3 + sex	H9	sex + date + date^2	9	3674.13	2.2	0.08	1843.78
		sex	H6	sex + date + date^2	8	3675.33	3.4	0.05	1847.02
		H2 + sex	H6	sex + date + date^2	9	3676.12	4.19	0.03	1845.76
		sex	.	sex + date + date^2	7	3677.27	5.34	0.02	1851.01
		H5 + sex	H6	sex + date + date^2	9	3677.32	5.39	0.02	1846.96
		H1 + sex	H6	sex + date + date^2	9	3677.37	5.44	0.02	1847.02

		sex	H7	sex + date + date^2	8	3677.4	5.47	0.02	1849.09
		sex	H8	sex + date + date^2	8	3677.84	5.91	0.01	1849.53
		H2 + sex	H7	sex + date + date^2	9	3678.21	6.28	0.01	1847.86
		sex	H9	sex + date + date^2	8	3678.68	6.75	0.01	1850.38
		H2 + sex	.	sex + date + date^2	8	3678.93	7	0.01	1850.62
		H1 + sex	.	sex + date + date^2	8	3678.98	7.05	0.01	1850.67
		H5 + sex	.	sex + date + date^2	8	3679.28	7.35	0.01	1850.97
		H5 + sex	H7	sex + date + date^2	9	3679.34	7.41	0.01	1848.98
		H1 + sex	H7	sex + date + date^2	9	3679.38	7.45	0.01	1849.03
		H2 + sex	H8	sex + date + date^2	9	3679.46	7.53	0.01	1849.1
		H1 + sex	H8	sex + date + date^2	9	3679.67	7.74	0.01	1849.32
20	*Rana luteiventris*	H2 + sex	H8	date	7	3366.19	0	0.2	3351.96
		sex	H8	date	6	3366.57	0.38	0.16	3354.4
		H2 + sex	H6	date	7	3367.36	1.17	0.11	3353.13
		H1 + sex	H8	date	7	3367.41	1.22	0.11	3353.18
		H2 + sex	.	date	6	3368.39	2.2	0.07	3356.22
		H5 + sex	H8	date	7	3368.43	2.24	0.06	3354.2
		H3 + sex	H8	date	7	3368.6	2.41	0.06	3354.37
		sex	H6	date	6	3368.86	2.68	0.05	3356.69
		H1 + sex	H6	date	7	3369.77	3.58	0.03	3355.54
		H2 + sex	H7	date	7	3370.16	3.97	0.03	3355.93
		H2 + sex	H9	date	7	3370.3	4.11	0.03	3356.07
		H5 + sex	H6	date	7	3370.48	4.29	0.02	3356.25
		H3 + sex	H6	date	7	3370.74	4.55	0.02	3356.51
		H1 + sex	.	date	6	3371.67	5.49	0.01	3359.5
		sex	.	date	5	3371.92	5.73	0.01	3361.79
		H5 + sex	.	date	6	3373.05	6.86	0.01	3360.88
21	*Anaxyrus boreas*	H3 + sex	H8 + sex	sex + date + date^2	10	27695.81	0	1	27675.75
22	*Rana cascadae*	H3 + sex	H7	sex + date + date^2	9	3948.06	0	0.46	1115.22
		H1 + sex	H7	sex + date + date^2	9	3950.35	2.3	0.15	1117.52
		H5 + sex	H7	sex + date + date^2	9	3951.03	2.97	0.1	1118.19

		sex	H7	sex + date + date^2	8	3951.79	3.73	0.07	1121
		H3 + sex	H7	sex + date + date^2	9	3952.46	4.41	0.05	1119.63
		H5 + sex	H8	sex + date + date^2	9	3952.75	4.69	0.04	1119.91
		H2 + sex	H8	sex + date + date^2	9	3953.08	5.02	0.04	1120.24
		sex	H8	sex + date + date^2	8	3953.12	5.06	0.04	1122.33
		H1 + sex	H8	sex + date + date^2	9	3954.12	6.06	0.02	1121.28
		H2 + sex	H8	sex + date + date^2	9	3955.03	6.98	0.01	1122.2
23	*Rana luteiventris*	H3 + sex	H9 + sex	date	8	26828.14	0	1	26812.11
24	*Rana sierrae*	H3 + sex	H7 + sex	sex + date + date^2	13	28737.8	0	0.98	28711.7
		H3 + sex	H9 + sex	sex + date + date^2	13	28745.56	7.76	0.02	28719.46
25	*Rana luteiventris*	H5	H8 + sex	sex + date + date^2	9	43169.45	0	1	43151.41
26	*Pseudacris maculata*	H5 + sex	sex	sex + date	8	13403.76	0	0.29	13387.68
		H5 + sex	H7 + sex	sex + date	9	13404.04	0.27	0.25	13385.93
		H5 + sex	H9 + sex	sex + date	9	13404.38	0.62	0.21	13386.27
		H5 + sex	H8 + sex	sex + date	9	13405.29	1.52	0.14	13387.18
		H5 + sex	H6 + sex	sex + date	9	13405.78	2.02	0.11	13387.68
27	*Anaxyrus boreas*	H1 + sex	H7 + sex	sex + date + date^2	10	8165.84	0	1	8145.63
28	*Anaxyrus boreas*	H2	H8	date + date^2	7	1413.47	0	0.12	1399.13
		.	H8	date + date^2	6	1413.68	0.2	0.11	1401.42
		H3	H8	date + date^2	7	1414.54	1.07	0.07	1400.2
		H2	.	date + date^2	6	1414.93	1.45	0.06	1402.67
		H5	H8	date + date^2	7	1414.98	1.51	0.06	1400.64
		H2	H9	date + date^2	7	1415.08	1.6	0.05	1400.73
		.	.	date + date^2	5	1415.22	1.75	0.05	1405.04
		.	H9	date + date^2	6	1415.39	1.92	0.05	1403.13
		H4	H8	date + date^2	7	1415.71	2.23	0.04	1401.36
		H1	H8	date + date^2	7	1415.76	2.29	0.04	1401.41
		H3	.	date + date^2	6	1415.96	2.49	0.03	1403.7

		H3	H9	date + date^2	7	1416.05	2.58	0.03	1401.71
		H2	H6	date + date^2	7	1416.35	2.88	0.03	1402.01
		H5	.	date + date^2	6	1416.42	2.95	0.03	1404.16
		H5	H9	date + date^2	7	1416.63	3.15	0.02	1402.28
		.	H6	date + date^2	6	1416.66	3.18	0.02	1404.4
		H2	H7	date + date^2	7	1416.84	3.37	0.02	1402.5
		.	H7	date + date^2	6	1417.09	3.61	0.02	1404.83
		H4	.	date + date^2	6	1417.25	3.78	0.02	1404.99
		H1	.	date + date^2	6	1417.29	3.82	0.02	1405.03
		H3	H6	date + date^2	7	1417.38	3.9	0.02	1403.03
		H4	H9	date + date^2	7	1417.46	3.98	0.02	1403.11
		H1	H9	date + date^2	7	1417.46	3.99	0.02	1403.12
		H5	H6	date + date^2	7	1417.87	4.4	0.01	1403.53
		H3	H7	date + date^2	7	1417.9	4.42	0.01	1403.55
		H5	H7	date + date^2	7	1418.3	4.83	0.01	1403.96
		H4	H6	date + date^2	7	1418.7	5.23	0.01	1404.36
		H1	H6	date + date^2	7	1418.74	5.26	0.01	1404.39
		H4	H7	date + date^2	7	1419.12	5.64	0.01	1404.77
		H1	H7	date + date^2	7	1419.17	5.7	0.01	1404.83
29	*Pseudacris maculata*	H1 + sex	H8	sex + date	8	27455.73	0	0.27	27439.68
		H1 + sex	.	sex + date	7	27455.87	0.14	0.25	27441.83
		H1 + sex	H7	sex + date	8	27455.95	0.22	0.24	27439.9
		H1 + sex	H9	sex + date	8	27457.38	1.65	0.12	27441.33
		H1 + sex	H6	sex + date	8	27457.64	1.92	0.1	27441.6
30	*Anaxyrus boreas*	H5	H9	date	6	5359.94	0	0.24	5347.85
		H2	H9	date	6	5360.15	0.21	0.22	5348.06
		H1	H9	date	6	5360.86	0.92	0.15	5348.77
		.	H9	date	5	5361.18	1.24	0.13	5351.12
		H4	H9	date	6	5362.99	3.04	0.05	5350.9
		H3	H9	date	6	5363.2	3.26	0.05	5351.11
		.	H6	date	5	5363.56	3.62	0.04	5353.5
		H5	H6	date	6	5363.82	3.88	0.03	5351.73

		H2	H6	date	6	5364.1	4.16	0.03	5352.01
		H3	H6	date	6	5364.21	4.27	0.03	5352.12
		H1	H6	date	6	5365.11	5.17	0.02	5353.02
		H4	H6	date	6	5365.52	5.58	0.01	5353.43
31	Anaxyrus boreas	H5 + sex	H6	sex + date + date^2	9	10863.81	0	0.8	10845.68
		H5 + sex	.	sex + date + date^2	8	10868.62	4.81	0.07	10852.52
		H5 + sex	H7	sex + date + date^2	9	10869.65	5.84	0.04	10851.52
		H5 + sex	H8	sex + date + date^2	9	10869.83	6.02	0.04	10851.7
		H5 + sex	H9	sex + date + date^2	9	10869.84	6.02	0.04	10851.71

Supplementary information 4: Summary: top hypotheses with beta estimates

A. SURVIVAL

Dataset (see Table 1)	Zone: Maritime=MAR Mediterranean=MED Montane=MON	Species	Elevation (m)	Survival :Top Hypothesis	AICc wt	Beta estimate	LCI	UCI	SE	Predicted direction of effect	Modelled direction of effect	Model result vs prediciton	R squared (amt of variance explained)
1	MAR	TRMA	122	Hypothesis 5: Recurrence of warm events	0.932	0.705	0.648	0.762	0.029	Neg	Pos	Contrary to	0.68
2	MAR	BOVA	137	Hypothesis 5: Recurrence of warm events	0.232	-0.319	-0.458	-0.180	0.070	Neg	Neg	As predicted	0.20
3	MED	TRCR	140	Hypothesis 4: Winter severity	0.995	-0.391	-0.408	-0.374	0.009	Neg	Neg	As predicted	0.10
4	MAR	BOVA	156	Hypothesis 4: Winter severity	0.170	0.046	0.039	0.053	0.004	Neg	Pos	Contrary to	0.14
5	MAR	ANFO	174	Hypothesis 4: Winter severity	0.817	-0.126	-0.131	-0.121	0.002	Neg	Neg	As predicted	0.21
6	MAR	TRCR	305	Hypothesis 5: Recurrence of warm events	1.000	0.360	0.356	0.364	0.002	Neg	Pos	Contrary to	0.38
7	MAR	SASA	450	Hypothesis 5: Recurrence of warm events	0.410	-0.317	-0.414	-0.220	0.050	Neg	Neg	As predicted	0.44
8	MAR	SASA	410	Hypothesis 4: Winter severity	0.347	-0.460	-0.728	-0.192	0.137	Neg	Neg	As predicted	0.14
9	MED	RAMU	859	Hypothesis 3: Lenghth of Winter	0.530	-2.594	-9.982	4.794	3.694	Neg	Neg	As predicted	0.28
10	MON	ANBO	1230	Hypothesis 5: Recurrence of warm events	0.988	0.835	0.814	0.856	0.010	Neg	Pos	Contrary to	0.46
11	MON	RACA	1293	Hypothesis 2: Unusual hot events	0.639	-0.034	-0.035	-0.033	0.000	Neg	Neg	As predicted	0.19
12	MED	RAMU	1547	Hypothesis 1: Drought	0.295	-8.347	-117.953	101.259	54.803	Neg	Neg	As predicted	0.39
13	MED	RAMU	1628	Hypothesis 5: Recurrence of warm events	0.318	-2.553	-16.311	11.205	6.879	Neg	Neg	As predicted	0.69
14	MED	RAMU	1643	Hypothesis 2: Unusual Hot events	0.632	-0.258	-0.292	-0.224	0.017	Neg	Neg	As predicted	0.30
15	MED	RAMU	1722	Hypothesis 5 : Recurrence of warm events	1.000	-10.584	-18.198	-2.970	3.885	Neg	Neg	As predicted	0.32
16	MON	RALU	1774	Hypothesis 3: Lenghth of Winter	1.000	-1.236	-1.266	-1.206	0.015	Neg	Neg	As predicted	0.55
17	MED	RAMU	1775	Hypothesis 0: Constant Survival	0.182	N/A	1.303	2.075	N/A	N/A	N/A	N/A	N/A
18	MED	RAMU	1804	Hypothesis 3: Lenghth of Winter	0.434	-1.487	-4.261	1.287	1.387	Neg	Neg	As predicted	0.29
19	MED	RAMU	1932	Hypothesis 3: Lenghth of Winter	0.249	-1.059	-2.599	0.481	0.770	Neg	Neg	As predicted	0.28
20	MON	RALU	1995	Hypothesis 2: Unusual Hot events	0.196	0.019	0.018	0.020	0.000	Neg	Pos	Contrary to	0.07
21	MON	ANBO	2085	Hypothesis 3: Lenghth of Winter	1.000	1.828	1.767	1.889	0.030	Neg	Pos	Contrary to	0.48
22	MON	RACA	2100	Hypothesis 3: Lenghth of Winter	0.464	-0.809	-1.348	-0.270	0.270	Neg	Neg	As predicted	0.27
23	MON	RALU	2200	Hypothesis 3: Lenghth of Winter	1.000	1.446	1.379	1.513	0.034	Neg	Pos	Contrary to	0.40
24	MON	RASI	2200	Hypothesis 3: Lenghth of Winter	0.980	-0.827	-0.893	-0.761	0.033	Neg	Neg	As predicted	0.54
25	MON	RALU	2478	Hypothesis 5: Recurrence of warm events	0.996	0.813	0.759	0.867	0.027	Neg	Pos	Contrary to	0.32
26	MON	PSMA	2803	Hypothesis 5: Recurrence of warm events	0.291	-0.304	-0.421	-0.187	0.059	Neg	Neg	As predicted	0.17
27	MON	ANBO	2810	Hypothesis 1: Drought	1.000	0.558	0.548	0.568	0.005	Neg	Pos	Contrary to	0.54
28	MON	ANBO	2946	Hypothesis 2: Unusual Hot events	0.119	0.016	0.015	0.017	0.001	Neg	Pos	Contrary to	0.21

29	MON	PSMA	2969	Hypothesis 1: Drought	0.270	-0.046	-0.049	-0.043	0.001	Neg	Neg	As predicted	0.10
30	MON	ANBO	3138	Hypothesis 5: Recurrence of warm events	0.239	0.077	0.054	0.100	0.012	Neg	Pos	Contrary to	0.09
31	MON	ANBO	3266	Hypothesis 5: Recurrence of warm events	0.805	-0.684	-0.887	-0.481	0.102	Neg	Neg	As predicted	0.48

B. RECRUITMENT

**Dataset (see Table 1)**	Zone: Maritime=MAR Mediterranean=MED Montane=MON	**Species**	**Elevation (m)**	**Recruitment: Top Hypothesis**	**AIC wt**	**Beta estimate**	**LCL**	**UCL**	**SE**	**Predicted direction of effect**	**Modelled direction of effect**	**Model result vs prediciton**	**R squared (amt of variance explained)**
1	MAR	TRMA	122	Hypothesis 9: Early winter	0.932	-0.013	-0.013	-0.013	0.000	Neg	Neg	As predicted	0.41
2	MAR	BOVA	137	Hypothesis 0: Constant recruitment	0.232	N/A	N/A	N/A	N/A	N/A	N/A	N/A	N/A
3	MED	TRCR	140	Hypothesis 6: Drought	0.995	0.083	0.083	0.083	0.000	Neg	Pos	Contrary to	0.40
4	MAR	BOVA	156	Hypothesis 8: Cold temperatures	0.17	-0.359	-0.507	-0.211	0.076	Neg	Neg	As predicted	0.35
5	MAR	ANFO	174	Hypothesis 7: Length of winter	0.817	0.909	0.582	1.236	0.167	Neg	Pos	Contrary to	0.30
6	MAR	TRCR	305	Hypothesis 8: Cold temperatures	1	0.064	0.064	0.064	0.000	Neg	Pos	Contrary to	0.23
7	MAR	SASA	450	Hypothesis 7: Length of winter	0.41	-0.402	-0.570	-0.234	0.086	Neg	Neg	As predicted	0.19
8	MAR	SASA	410	Hypothesis 9: Early winter	0.347	0.005	0.005	0.005	0.000	Neg	Pos	Contrary to	0.05
9	MED	RAMU	859	Hypothesis 8: Cold temperatures	0.53	0.186	0.160	0.212	0.013	Neg	Pos	Contrary to	0.14
10	MON	ANBO	1230	Hypothesis 7: Length of winter	0.988	2.45	2.169	2.731	0.143	Neg	Pos	Contrary to	0.33
11	MON	RACA	1293	Hypothesis 9: Early winter	0.639	-0.011	-0.011	-0.011	0.000	Neg	Neg	As predicted	0.06
12	MED	RAMU	1547	Hypothesis 9: Early winter	0.295	0.011	0.011	0.011	0.000	Neg	Pos	Contrary to	0.69
13	MED	RAMU	1628	Hypothesis 8: Cold temperatures	0.318	0.197	0.009	0.385	0.096	Neg	Pos	Contrary to	0.29
14	MED	RAMU	1643	Hypothesis 6: Drought	0.632	0.004	0.004	0.004	0.000	Neg	Pos	Contrary to	0.14
15	MED	RAMU	1722	Hypothesis 8 : Cold Temperatures	1	-0.44205	-0.782	-0.102	0.173	Neg	Neg	As predicted	0.50
16	MON	RALU	1774	Hypothesis 6: Drought	1	-0.013	-0.013	-0.013	0.000	Neg	Neg	As predicted	0.16
17	MED	RAMU	1775	Hypothesis 9: Early winter	0.182	-0.017	-0.017	-0.017	0.000	Neg	Neg	As predicted	0.53
18	MED	RAMU	1804	Hypothesis 8: Cold temperatures	0.434	0.289	0.201	0.377	0.045	Neg	Pos	Contrary to	0.44
19	MED	RAMU	1932	Hypothesis 6: Drought	0.249	0.001	0.001	0.001	0.000	Neg	Pos	Contrary to	0.06
20	MON	RALU	1995	Hypothesis 8 : Cold temperatures	0.196	-0.657	-1.415	0.101	0.387	Neg	Neg	As predicted	0.15
21	MON	ANBO	2085	Hypothesis 8 : Cold temperatures	1	-0.184	-0.185	-0.183	0.000	Neg	Neg	As predicted	0.29
22	MON	RACA	2100	Hypothesis 7: Length of winter	0.464	0.988	0.095	1.881	0.455	Neg	Pos	Contrary to	0.36
23	MON	RALU	2200	Hypothesis 9: Early winter	1	-0.023	-0.023	-0.023	0.000	Neg	Neg	As predicted	0.22
24	MON	RASI	2200	Hypothesis 7: Length of winter	0.98	-0.158	-0.160	-0.156	0.001	Neg	Neg	As predicted	0.13
25	MON	RALU	2478	Hypothesis 8: Cold temperatures	0.996	1.138	1.122	1.154	0.008	Neg	Pos	Contrary to	0.39
26	MON	PSMA	2803	Hypothesis 0: Constant recruitment	0.291	N/A	N/A	N/A	N/A	N/A	N/A	N/A	N/A

27	MON	ANBO	2810	Hypothesis 7: Length of winter	1	-0.965	-0.979	-0.951	0.007	Neg	Neg	As predicted	0.46
28	MON	ANBO	2946	Hypothesis 8: Cold temperatures	0.119	0.194	0.126	0.262	0.035	Neg	Pos	Contrary to	0.37
29	MON	PSMA	2969	Hypothesis 8: Cold temperatures	0.27	0.006	0.006	0.006	0.000	Neg	Pos	Contrary to	0.01
30	MON	ANBO	3138	Hypothesis 9 : Early winter	0.239	0.046	0.044	0.048	0.001	Neg	Pos	Contrary to	0.28
31	MON	ANBO	3266	Hypothesis 6: Drought	0.805	-0.029	-0.029	-0.029	0.000	Neg	Neg	As predicted	0.08

Supplementary information 5: R squared values - hypotheses, survival and recruitment (by zone, taxa, mode of hibernation)																					
			R square = Proportion variance explained									Parameter (log-odds coefficient) estimates									
			HYPOTHESES - SURVIVAL					HYPOTHESES - RECRUITMENT				HYPOTHESES - SURVIVAL					HYPOTHESES - RECRUITMENT				
data set	zone		H1	H2	H3	H4	H5	H6	H7	H8	H9	H1	H2	H3	H4	H5	H6	H7	H8	H9
1	MAR	TRMA	0.54	0.33	0.08	0.05	0.68	0.00	0.07	0.02	0.41	0.00	0.00	0.00	0.00	0.71	0.00	0.00	0.00	-0.01
2	MAR	BOVA	0.04	0.01	0.03	0.00	0.20	0.10	0.10	0.01	0.10	0.00	0.00	0.00	0.00	-0.32	0.00	0.08	-0.01	0.00
3	MED	TRCR	0.03	0.02	0.01	0.10	0.01	0.40	0.09	0.01	0.13	0.00	0.00	0.00	-0.39	0.00	0.08	0.00	0.00	0.00
4	MAR	BOVA	0.01	0.05	0.02	0.14	0.11	0.28	0.26	0.35	0.00	0.00	0.00	-0.04	0.05	0.03	0.00	0.12	-0.36	0.00
5	MAR	ANFO	0.01	0.02	0.08	0.21	0.00	0.13	0.30	0.01	0.01	0.00	0.00	-0.07	-0.13	0.00	0.00	0.91	0.00	0.00
6	MAR	TRCR	0.00	0.02	0.04	0.00	0.38	0.01	0.07	0.23	0.09	0.00	0.00	0.00	0.00	0.36	0.00	0.00	0.06	0.00
7	MAR	SASA	0.06	0.03	0.25	0.02	0.44	0.01	0.19	0.02	0.11	0.00	0.00	-0.28	0.00	-0.32	0.00	-0.40	0.00	0.00
8	MAR	SASA	0.00	0.00	0.00	0.14	0.01	0.02	0.03	0.03	0.05	0.00	0.00	0.00	-0.46	0.00	0.00	-0.09	0.01	0.01
9	MED	RAMU	0.01	0.11	0.28	NA	0.05	0.04	0.01	0.14	0.07	0.00	-0.01	-2.59	0.00	0.01	0.00	-0.01	0.19	0.00
10	MON	ANBO	0.04	0.42	0.04	0.30	0.46	0.01	0.33	0.20	0.12	0.00	0.00	0.00	0.00	0.84	0.00	2.45	0.00	0.00
11	MON	RACA	0.00	0.19	0.03	NA	0.10	0.04	0.01	0.01	0.06	0.00	-0.03	0.00	0.00	0.00	0.00	0.00	0.00	-0.01
12	MED	RAMU	0.39	0.01	0.34	NA	0.01	0.66	0.52	0.04	0.69	-7.27	0.00	0.00	0.00	0.00	0.00	-0.03	-0.01	0.01
13	MED	RAMU	0.20	0.02	0.14	NA	0.69	0.26	0.18	0.29	0.16	0.00	0.00	0.00	0.00	-2.55	-0.01	0.18	0.20	-0.01
14	MED	RAMU	0.16	0.30	0.04	NA	0.08	0.14	0.05	0.02	0.07	0.00	-0.26	0.02	0.00	-0.02	0.00	0.03	0.00	0.00
15	MED	RAMU	0.02	0.03	0.09	NA	0.32	0.17	0.03	0.50	0.04	0.00	0.00	0.00	0.00	-10.58	0.00	0.00	-0.44	0.00
16	MON	RALU	0.02	0.01	0.55	NA	0.44	0.16	0.02	0.02	0.01	0.00	0.00	-1.24	0.00	0.00	-0.01	0.00	0.00	0.00
17	MED	RAMU	0.06	0.05	0.06	NA	0.01	0.06	0.51	0.01	0.53	0.00	0.04	-0.20	0.00	0.01	0.00	0.40	0.00	-0.02
18	MED	RAMU	0.00	0.24	0.29	NA	0.02	0.01	0.11	0.44	0.07	0.00	0.26	-1.49	0.00	-0.01	0.00	-0.05	0.29	0.00
19	MED	RAMU	0.00	0.06	0.28	NA	0.00	0.06	0.02	0.03	0.01	0.00	-0.01	-1.06	0.00	0.00	0.00	-0.02	-0.01	0.00
20	MON	RALU	0.04	0.07	0.00	NA	0.01	0.11	0.01	0.15	0.01	0.00	0.02	-0.01	0.00	0.01	0.00	0.00	-0.66	0.00
21	MON	ANBO	0.01	0.05	0.48	0.00	0.35	0.22	0.14	0.29	0.07	0.00	0.00	1.83	0.00	0.00	0.00	0.00	-0.18	0.00
22	MON	RACA	0.16	0.06	0.27	NA	0.13	0.04	0.36	0.25	0.00	0.00	0.00	-0.81	0.00	0.02	0.00	0.99	-0.01	0.00
23	MON	RALU	0.03	0.05	0.40	NA	0.03	0.00	0.02	0.01	0.22	0.00	0.00	1.45	0.00	0.00	0.00	0.00	0.00	-0.02
24	MON	RASI	0.12	0.02	0.54	NA	0.16	0.02	0.13	0.03	0.09	0.00	0.00	-0.83	0.00	0.00	0.00	-0.16	0.00	0.00
25	MON	RALU	0.24	0.15	0.04	NA	0.32	0.20	0.32	0.39	0.20	0.00	0.00	0.00	0.00	0.81	0.00	0.00	1.14	0.00
26	MON	PSMA	0.02	0.01	0.02	0.05	0.17	0.00	0.01	0.00	0.01	0.00	0.00	0.00	0.00	-0.30	0.00	-0.02	0.00	0.00
27	MON	ANBO	0.54	0.41	0.02	0.34	0.33	0.07	0.46	0.22	0.12	0.56	0.00	0.00	0.00	0.00	0.00	-0.97	0.00	0.00
28	MON	ANBO	0.00	0.21	0.11	0.01	0.07	0.07	0.02	0.37	0.20	0.00	0.02	0.25	0.01	0.03	-0.01	0.02	0.19	0.01
29	MON	PSMA	0.10	0.02	0.00	0.00	0.02	0.00	0.01	0.01	0.00	-0.05	0.00	0.00	0.00	0.00	0.00	-0.01	0.01	0.00
30	MON	ANBO	0.07	0.09	0.00	0.01	0.09	0.22	0.01	0.07	0.28	0.01	0.01	-0.01	0.00	0.08	0.01	0.00	0.00	0.05
31	MON	ANBO	0.06	0.15	0.19	0.02	0.48	0.08	0.01	0.01	0.01	0.00	0.00	0.00	0.00	-0.68	-0.03	-0.02	0.00	0.00

SURVIVAL BY ZONE						
	dataset	species	r^2	average r^2 for hypothesis (predicted or contrary)		
**MARITIME n = 7**						
**H4**						
As predicted	5	ANFO	0.210	0.175		
	8	SASA	0.140			
Contrary to	4	BOVA	0.140	0.140		
**H5**						
As predicted	2	BOVA	0.200	0.320		
	7	SASA	0.440			
Contrary to	1	TRMA	0.680	0.530		
	6	TRCR	0.380			
**MEDITERRANEAN n = 9**						
**HO**	17		n/a			
**H1**						
As predicted	12	RAMU	0.390			
Contrary to	--					
**H2**						
As predicted	14	RAMU	0.300			
Contrary to	--					
**H3**						
As predicted	9	RAMU	0.280	0.283		
	18	RAMU	0.290			
	19	RAMU	0.280			
Contrary to	--					
**H4**						
As predicted	3	TRCR	0.100			
Contrary to	--					
**H5**						
As predicted	13	RAMU	0.690	0.505		
	15	RAMU	0.320			
Contrary to						
**MONTANE n = 15**						
**H1**						
As predicted	29	PSMA	0.100			
Contrary to	27	ANBO	0.540			
**H2**						
As predicted	11	RACA	0.190			
Contrary to	20	RALU	0.070	0.140		
	28	ANBO	0.210			
**H3**						
As predicted	16	RALU	0.550	0.453		
	24	RASI	0.540			
	22	RACA	0.270			
Contrary to	21	ANBO	0.480	0.220		
	23	RALU	0.400			
**H5**						

As predicted	26	PSMA	0.170	0.325		
	31	ANBO	0.480			
Contrary to	10	ANBO	0.460	0.290		
	25	RALU	0.320			
	30	ANBO	0.090			
**SURVIVAL BY TAXA**						
**RANID FROGS n = 15**						
**H0**	17	RAMU	n/a			
**H1**						
As predicted	12	RAMU	0.39			
Contrary to	--					
**H2**						
As predicted	14	RAMU	0.30	0.245		
	11	RACA	0.19			
Contrary to	20	RALU	0.07			
**H3**						
As predicted	9	RAMU	0.28	0.368		
	18	RAMU	0.29			
	19	RAMU	0.28			
	16	RALU	0.55			
	24	RASI	0.54			
	22	RACA	0.27			
Contrary to	23	RALU	0.40			
**H5**						
As predicted	13	RAMU	0.69	0.505		
	15	RAMU	0.32			
Contrary to	25	RALU				
**TREEFROGS n = 2**						
**H1**						
As predicted	29	PSMA	0.10			
Contrary to	--					
**H5**						
As predicted	26	PSMA	0.17			
Contrary to	--					
**TOADS n = 9**						
**H1**						
As predicted	--					
Contrary to	27	ANBO	0.54			
**H2**						
As predicted	--					
Contrary to	28	ANBO	0.21			
**H3**						
As predicted	--					
Contrary to	21	ANBO	0.48			
**H4**						
As predicted	5	ANFO	0.21			
Contrary to	4	BOVA	0.00			
**H5**						
As predicted	2	BOVA	0.11	0.285		

	31	ANBO	0.46			
Contrary to	10	ANBO	0.09	0.285		
	30	ANBO	0.48			
**NEWTS / SALAMANDERS n = 5**						
**H4**						
As predicted	8	SASA	0.14	0.120		
	3	TRCR	0.10			
Contrary to	--					
**H5**						
As predicted	7	SASA	0.44			
Contrary to	1	TRMA	0.68	0.530		
	6	TRCR	0.38			
**SURVIVAL BY HIBERNATION MODE**						
**TERRESTRIAL n = 15**						
**H1**						
As predicted	29	PSMA	0.10			
Contrary to	27	ANBO	0.54			
**H2**						
As predicted	--					
Contrary to	28	ANBO				
**H3**						
As predicted	--					
Contrary to	21	ANBO	0.48			
**H4**						
As predicted	5	ANFO	0.21	0.150		
	8	SASA	0.14			
	3	TRCR	0.10			
Contrary to	4	BOVA	0.00			
**H5**						
As predicted	26	PSMA	0.17	0.300		
	31	ANBO	0.48			
	2	BOVA	0.11			
	7	SASA	0.44			
Contrary to	1	TRMA	0.68	0.403		
	6	TRCR	0.38			
	30	ANBO	0.09			
	10	ANBO	0.46			
**AQUATIC n = 15**						
**H0**	17	RAMU	n/a			
**H1**						
As predicted	12	RAMU	0.39			
Contrary to	--					
**H2**						
As predicted	14	RAMU	0.30	0.300		
	11	RACA	0.19			
Contrary to	20	RALU	0.07			
**H3**						
As predicted	9	RAMU	0.28	0.368		
	18	RAMU	0.29			

	19	RAMU	0.28			
	16	RALU	0.55			
	24	RASI	0.54			
	22	RACA	0.27			
Contrary to	23	RALU	0.40			
**H5**						
As predicted	13	RAMU	0.69	0.505		
	15	RAMU	0.32			
Contrary to	25	RALU	0.32			

RECRUITMENT BY ZONE						
	dataset	species	r^2	average r^2 for hypothesis (predicted or contrary)		
**MARITIME n = 7**						
**H0**	2	BOVA	n/a			
**H7**						
As predicted	7	SASA	0.19			
Contrary to	5	ANFO	0.3			
**H8**						
As predicted	4	BOVA	0.35			
Contrary to	6	TRCR	0.23			
**H9**						
As predicted	1	TRMA	0.41			
Contrary to	8	SASA	0.05			
**MEDITERRANEAN n = 9**						
**H6**						
As predicted	--					
Contrary to	3	TRCR	0.4	0.20		
	14	RAMU	0.14			
	19	RAMU	0.06			
**H8**						
As predicted	15	RAMU	0.5			
Contrary to	9	RAMU	0.14	0.29		
	13	RAMU	0.29			
	18	RAMU	0.44			
**H9**						
As predicted	17	RAMU	0.53			
Contrary to	12	RAMU	0.69			
**MONTANE n = 15**						
**H0**	26	PSMA	n/a			
**H6**						
As predicted	16	RALU	0.16	0.12		
	31	ANBO	0.08			
Contrary to	--					
**H7**						
As predicted	24	RASI	0.13	0.30		
	27	ANBO	0.46			
Contrary to	10	ANBO	0.33	0.35		
	22	RACA	0.36			
**H8**						
As predicted	20	RALU	0.15	0.22		
	21	ANBO	0.29			
Contrary to	25	RALU	0.39	0.26		
	28	ANBO	0.37			
	29	PSMA	0.01			
**H9**						
As predicted	11	RACA	0.06	0.14		

	23	RALU	0.22			
Contrary to	30	ANBO	0.28			
**RECRUITMENT BY TAXA**						
**RANID FROGS = 15**						
**H6**						
As predicted	16	RALU	0.16			
Contrary to	14	RAMU	0.14	0.10		
	19	RAMU	0.06			
**H7**						
As predicted	24	RASI	0.13			
Contrary to	22	RACA	0.36			
**H8**						
As predicted	20	RALU	0.15	0.33		
	15	RAMU	0.5			
Contrary to	9	RAMU	0.14	0.32		
	13	RAMU	0.29			
	18	RAMU	0.44			
	25	RALU	0.39			
**H9**						
As predicted	17	RAMU	0.53	0.27		
	11	RACA	0.06			
	23	RALU	0.22			
Contrary to	12	RAMU	0.69			
**TREEFROGS n = 2**						
**H0**	26	PSMA	n/a			
**H8**						
As predicted	29	PSMA	0.01			
Contrary to	--					
**TOADS n = 9**						
**H0**	2	BOVA				
**H6**						
As predicted	31	ANBO	0.08			
Contrary to	--					
**H7**						
As predicted	27	ANBO	0.46			
Contrary to	5	ANFO	0.3	0.32		
	10	ANBO	0.33			
**H8**						
As predicted	21	ANBO	0.29	0.32		
	4	BOVA	0.35			
Contrary to	28	ANBO	0.37			
**H9**						
As predicted						
Contrary to	30	ANBO	0.28			
**NEWTS / SALAMANDERS n = 5**						
**H6**						

As predicted	--					
Contrary to	3	TRCR	0.4			
**H7**						
As predicted	7	SASA	0.19			
Contrary to	--					
**H8**						
As predicted	6	TRCR	0.23			
Contrary to	--					
**H9**						
As predicted	1	TRMA	0.41			
Contrary to	8	SASA	0.05			
**RECRUITMENT BY HIBERNATION MODE**						
**TERRESTRIAL n = 16**						
**H0**						
	2	BOVA	N/A			
	26	PSMA	N/A			
**H6**						
As predicted	31	ANBO	0.08			
Contrary to	3	TRCR	0.4			
**H7**						
As predicted	7	SASA	0.19	0.33		
	27	ANBO	0.46			
Contrary to	5	ANFO	0.3	0.32		
	10	ANBO	0.33			
**H8**						
As predicted	21	ANBO	0.29	0.32		
	4	BOVA	0.35			
Contrary to	6	TRCR	0.23	0.20		
	28	ANBO	0.37			
	29	PSMA	0.01			
**H9**						
As predicted	1	TRMA	0.41			
Contrary to	30	ANBO	0.28	0.17		
	8	SASA	0.05			
**AQUATIC n = 15**						
**H6**						
As predicted	16	RALU	0.16			
Contrary to	14	RAMU	0.14	0.10		
	19	RAMU	0.06			
**H7**						
As predicted	24	RASI	0.13			
Contrary to	22	RACA	0.36			
**H8**						
As predicted	20	RALU	0.15	0.33		
	15	RAMU	0.5			

Contrary to	9	RAMU	0.14	0.32		
	13	RAMU	0.29			
	18	RAMU	0.44			
	16	RALU	0.39			
**H9**						
As predicted	17	RAMU	0.53	0.27		
	11	RACA	0.06			
	23	RALU	0.22			
Contrary to	12	RAMU	0.69			

Supplementary information 6: Sources for covariate values by hypothesis; natural history information sources by species; hypothesis development detail.

		HYPOTHESIS 1			HYPOTHESIS 2		HYPOTHESIS 3	HYPOTHESIS 4		
Species	DataSet	Threshold temperature winter (^oC)	Threshold (number of drought days)	Coefficient of variation for number of drought days	Threshold warm temperatures (^oC)	Coefficient of variation for warm days	Coefficient of variation for length of winter	Threshold cold temperatures (^oC)	Length cold event (days)	Coefficient of variation for winter severity
Triturus marmoratus	1	0	7	1.088	27.055	0.486	0.197	-2.314	4.000	1.062
Bombina variegata	2	0	10	1.005	29.665	0.412	0.306	-1.740	5.000	1.327
Triturus cristatus	3	0	21	1.027	33.010	0.555	0.308	-0.391	5.000	1.212
Bombina variegata	4	0	10	1.005	29.665	0.412	0.306	-1.740	5.000	1.327
Anaxyrus fowleri	5	0	11	1.170	30.586	1.121	0.172	-11.795	3.000	1.189
Triturus cristatus	6	0	12	1.019	31.245	0.576	0.281	-3.605	3.000	1.085
Salamandra salamandra	7	-4.44	7	1.214	22.540	0.647	0.192	-8.304	6.000	1.022
Salamandra salamandra	8	-4.44	7	1.071	22.540	0.669	0.198	-8.304	5.000	1.018
Rana muscosa	9	0	45	0.949	31.050	0.315	0.250	NA	NA	NA
Anaxyrus boreas	10	-4.44	15	1.005	33.539	0.649	0.155	-16.264	6.000	1.656
Rana cascadae	11	0	15	1.075	22.587	0.410	0.210	NA	NA	NA
Rana muscosa	12	0	34	1.029	29.712	0.355	0.217	NA	NA	NA
Rana muscosa	13	0	36	1.003	32.558	0.394	0.210	NA	NA	NA
Rana muscosa	14	0	35	1.031	34.951	0.407	0.267	NA	NA	NA
Rana muscosa	15	0	45	0.990	33.338	0.549	0.233	NA	NA	NA
Rana luteiventris	16	-4.44	26	1.039	33.961	0.976	0.256	NA	NA	NA
Rana muscosa	17	0	33	1.074	32.028	1.325	0.222	NA	NA	NA
Rana muscosa	18	0	30	1.034	32.028	1.414	0.213	NA	NA	NA
Rana muscosa	19	0	34	1.032	32.907	0.429	0.248	NA	NA	NA
Rana luteiventris	20	-4.44	14	1.012	30.978	0.717	0.183	NA	NA	NA
Anaxyrus boreas	21	-4.44	15	1.114	27.914	0.855	0.190	-21.378	3.000	1.079
Rana cascadae	22	-4.44	45	0.949	28.911	0.606	0.214	NA	NA	NA
Rana luteiventris	23	-4.44	13	1.052	29.167	0.678	0.178	NA	NA	NA
Rana Sierrae	24	0	44	0.998	27.600	0.616	0.227	NA	NA	NA
Rana luteiventris	25	-4.44	19	1.113	25.721	0.605	0.191	NA	NA	NA
Pseudacris maculata	26	-4.44	14	1.113	24.694	0.716	0.161	-19.306	4.000	1.160
Anaxyrus boreas	27	-4.44	12	0.997	26.998	0.803	0.172	-16.470	4.000	1.084
Anaxyrus boreas	28	-4.44	10	1.018	26.595	0.815	0.175	-16.013	5.000	1.809
Pseudacris maculata	29	-4.44	10	1.097	24.075	0.707	0.158	-19.363	4.000	1.131
Anaxyrus boreas	30	-4.44	9	1.083	21.478	0.767	0.240	-21.197	4.000	1.519
Anaxyrus boreas	31	-4.44	10	1.189	25.311	0.649	0.180	-17.837	4.000	1.089

HYPOTHESIS 5			HYPOTHESIS 6	HYPOTHESIS 7	HYPOTHESIS 8		HYPOTHESIS 9	
Threshold warm temperatures (^{o}C)	Threshold (number of warm days)	Coefficient of variation for warm days during hibernation	Coefficient of variation for drought days	Coefficient of variation length of winter	Cold threshold (^{o}C)	Coefficient of variation for cold relative to egg laying	Julien date	Coefficient of variation for onset of winter
10.917	3.000	1.144	0.550	0.092	2.037	0.551	4	0.338
11.544	1.000	1.103	0.599	0.191	3.886	1.308	2	0.381
12.091	1.000	1.758	0.657	0.252	-0.180	1.244	4	0.759
11.544	1.000	1.103	0.599	0.191	3.886	1.308	2	0.381
10.162	1.000	1.555	0.669	0.145	3.197	2.356	306	0.853
10.628	1.000	1.303	0.801	0.200	3.257	0.608	2	0.415
6.217	1.000	1.741	0.688	0.127	-5.587	0.791	305	0.478
6.217	1.000	1.795	0.662	0.122	-5.587	0.864	305	0.568
12.460	5.000	1.103	0.334	0.116	1.196	0.845	278	0.508
6.441	1.000	1.291	0.561	0.075	-1.327	0.765	279	0.625
6.795	1.000	1.037	0.660	0.153	2.540	0.638	277	0.560
13.100	1.000	1.172	0.663	0.124	-0.080	0.695	275	0.860
12.479	6.000	1.252	0.571	0.126	1.335	0.690	275	0.697
14.262	3.000	1.297	0.644	0.135	2.974	0.701	285	0.488
11.648	5.000	1.064	0.470	0.137	3.388	0.787	15	0.292
8.956	1.000	1.380	0.770	0.242	-5.670	1.210	286	0.673
9.578	6.000	1.129	0.745	0.141	0.607	1.054	278	0.551
9.578	6.000	1.216	0.583	0.145	0.607	1.038	278	0.525
12.820	4.000	1.025	0.619	0.138	1.008	0.663	285	0.699
5.611	3.000	1.337	0.426	0.110	-1.320	3.464	282	0.504
5.263	1.000	1.522	0.509	0.150	-4.360	0.744	275	0.617
4.116	1.000	1.713	0.280	0.085	3.266	0.614	315	0.599
6.944	1.000	1.264	0.410	0.108	2.080	0.427	282	0.595
10.410	4.000	1.006	0.150	0.115	-3.060	1.482	275	0.830
5.746	1.000	1.323	0.485	0.115	-1.420	0.583	274	0.598
6.773	1.000	1.927	0.684	0.107	-10.680	1.139	264	0.631
6.140	1.000	1.049	0.627	0.118	-3.760	2.251	264	0.585
6.102	3.000	1.094	0.328	0.137	0.760	0.667	268	0.577
6.497	1.000	2.015	0.656	0.099	-1.520	0.480	264	0.650
4.886	1.000	1.826	0.534	0.148	-6.710	0.690	251	0.559
5.173	3.000	1.373	0.704	0.117	-0.320	0.636	264	0.617

1. Date sources temperature & precipitation / snowpack:

Canada

http://climate.weather.gc.ca/

local weather stations: Port Colborne weather station (42° 53' N, 79° 15' W; Climate ID: 6136606);

Long Point weather station (42° 33’ N, 80° 03’ W; Climate ID: 6134F10)

France

Meteo France (http://www.meteofrance.com/)

Germany

ftp://ftp-cdc.dwd.de/pub/CDC/observations_germany/climate/

http://www.dwd.de/DE/Home/home_node.html

local weather station: Kahler Asten, district of Hochsauerland, North Rhine-Westphalia

USA

PRISM (http://www.prism.oregonstate.edu/)

SNOTEL (http://www.wcc.nrcs.usda.gov/snow/

2. Published demographic references: ANBO - *A. boreas*, ANFO - *Anaxyrus fowleri*, BOVA - *Bombina variegata*, PSMA - *Pseudacris maculata*, RACA - *R. cascadae*, RALU - *R. lutieventris*, RAMU - *Rana muscosa*, RASI - *R. sierrae*. SASA - *Salamandra salamandra*, TRCR - *Triturus cristatus*, TRMA - *T. marmoratus*,

ANBO

Muths, E., P. S. Corn, A. P. Pessier, and D. E. Green. 2003. Evidence for disease-related amphibian decline in Colorado. Biological Conservation 110:357–365.

Pilliod, D.S., Muths, E., Scherer, R.D., Bartelt, P.E., Corn, P.S., Hossack, B.R., Lambert, B.A., Gaughan, C., 2010. Effects of amphibian chytrid fungus on individual survival probability in wild boreal toads. Conservation Biology, 24(5), pp.1259-1267.

Muths, E., Scherer, R.D. and Pilliod, D.S., 2011. Compensatory effects of recruitment and survival when amphibian populations are perturbed by disease. Journal of Applied Ecology, 48(4), pp.873-879.

ANFO

Green, D.M., 2003. The ecology of extinction: population fluctuation and decline in amphibians. Biological conservation, 111(3), pp.331-343.

Greenberg, D.A. and D.M. Green. 2013. Effects of an invasive plant on population dynamics in toads. Conservation Biology 27:1049-1057.

BOVA

Cayuela, H., Arsovski, D., Thirion, J.M., Bonnaire, E., Pichenot, J., Boitaud, S., Miaud, C., Joly, P. and Besnard, A., 2016a. Demographic responses to weather fluctuations are context dependent in a long-lived amphibian. Global Change Biology, 22, 2676–2687.

Cayuela, H., Arsovski, D., Bonnaire, E., Duguet, R., Joly, P. and Besnard, A., 2016b. The impact of severe drought on survival, fecundity, and population persistence in an endangered amphibian. Ecosphere, 7(2): e01246.

PSMA

Muths, E., Scherer, R.D., Amburgey, S.M., Matthews, T., Spencer, A.W. and Corn, P.S. 2016. First Estimates of the Probability of Survival in a Small-bodied, High Elevation Frog or, how Historical Data Can Be Useful. *Canadian Journal of Zoology*, 94(9): 599-606, 10.1139/cjz-2016-0024.

RACA

Garwood, J.M. and H.H. Welsh, Jr. 2007. Ecology of the Cascades Frog (*Rana cascadae*) and interactions with garter snakes (*Thamnophis spp.*) and nonnative trout in the Trinity Alps Wilderness, California. Final report prepared for the California Department of Fish and Game and the National Fish and Wildlife Foundation. Arcata CA. 87p.

RAMU

Backlin, A.R., Hitchcock, C., Gallegos, E., Yee, J., and R.N. Fisher. 2013. The precarious persistence of the endangered Sierra Madre yellow-legged frog (*Rana muscosa*) in southern California. *Oryx – The International Journal of Conservation* doi:10.1017/S003060531300029X

RALU

McCaffery, R.M. and Maxell, B.A., 2010. Decreased winter severity increases viability of a montane frog population. Proceedings of the National Academy of Sciences, 107(19), pp.8644-8649.

McCaffery, R.M., Eby, L.A., Maxell, B.A. and Corn, P.S., 2014. Breeding site heterogeneity reduces variability in frog recruitment and population dynamics. Biological Conservation, 170, pp.169-176.

Pilliod, D. S., & Scherer, R. D. (2015). Managing habitat to slow or reverse population declines of the Columbia spotted frog in the northern Great Basin. The Journal of Wildlife Management, 79(4), 579-590.

RASI

Fellers, G.M., Kleeman, P.M., Miller, D.A., Halstead, B.J. and Link, W.A. (2013). Population size, survival, growth, and movements of Rana sierrae. Herpetologica, 69,147-162.

SASA

Schmidt, B.R., Feldmann, R. and Schaub, M. (2005). Demographic processes underlying population growth and decline in *Salamandra salamandra*. *Cons. Biol.* **19**, 1149-1156.

TRCR, TRMA

Jehle, R., Arntzen, J.W., Burke, T., Krupa, A.P. and Hödl, W., 2001. The annual number of breeding adults and the effective population size of syntopic newts (Triturus cristatus, T. marmoratus). Molecular Ecology, 10(4), pp.839-850.

To assess survival, we hypothesized that:

1) adult survival is reduced by lack of water during active non-breeding season (H1) because of potential for physiological stress (Bartelt et al. 2004), lower food availability (Williams 1951) and general habitat degradation (pond drying, Amburgey et al. 2012, Hossack et al. 2013, Pilliod and Scherer 2015);

2) Unusually high temperatures can influence survival negatively due to desiccation and related lack of water (Rittenhouse et al. 2015) (H2);

3) Longer winters decrease time available for an individual to be active and reduce survival (e.g., emerging from long hibernation in weakened state; or reduced opportunities for foraging) (Carey et al. 2005,) (H3);

4) In montane habitats, exposure to cold temperatures reduce survival (H4). However, cold experienced by hibernating animals is buffered by snowpack; in low snowpack years, cold temperatures may have greater impact. Thus, snowpack (as represented by a measure of snow water equivalent [SWE]) was accounted for in developing covariate H4 (O’Connor and Rittenhouse 2016);

5) Bouts of unseasonably warm temperatures during hibernation (i.e., winter) cause physiological arousal (Sinclair et al. 2013), interrupt hibernation, waste energy, and reduce survival (H5). Snowpack also buffers against warm temperatures so we accounted for the effect of snowpack during the bouts of warm weather in winter.

To assess recruitment we hypothesized that:

1) Recruitment is reduced by lack of water due to increased desiccation risk to small bodied juveniles, and reduced food and habitat (H6);

2) Longer and colder winters decrease the amount of time available for an individual to be active and will reduce survival and thus recruitment (H7);

3) Cold temperatures in spring that result in damage or destruction of eggs will reduce recruitment (Håkansson and Loman 2004) (H8); and

4) Freezing events in the autumn before metamorphic animals have successfully left the breeding site will reduce recruitment; in other words, if the onset of winter (i.e., freezing events) is later, recruitment will be influenced positively (H9).

Supplementary information 7: Average estimated lambda values by zone (maritime, Mediterranean, montane)

MARITIME

Average estimated lambda values: black dashed line=males; grey line=females; black hash & dot line=males&females; X axis indicates yr in dataset. Values >1 indicate positive population growth rate; values <1 indicate negative population growth rate. Numbers indicate datasets. Note: scale is NOT the same on all graphs. Species: ANFO-*Anaxyrus* fowleri, BOVA – *Bombina variegata* , SASA – *Salamandra salamandra* , TRCR – Triturus cristatus, TRMA – *T. marmoratus* .

TRMA lambda=1.00, (CI=0.92-1.09) 1

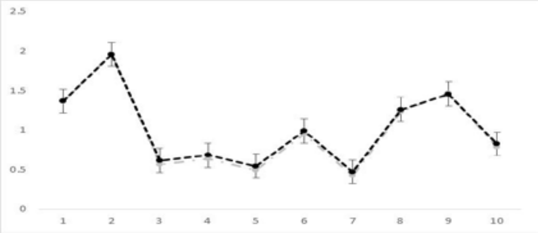

BOVA lambda=0.96, (CI=0.88-1.02) 2

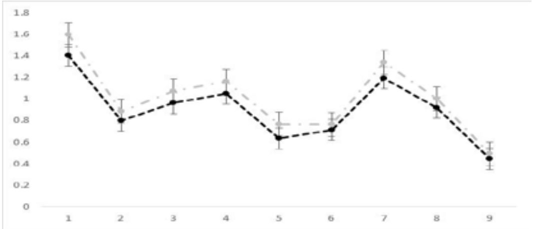

BOVA lambda=1.08, (CI=1.02-1.14) 4

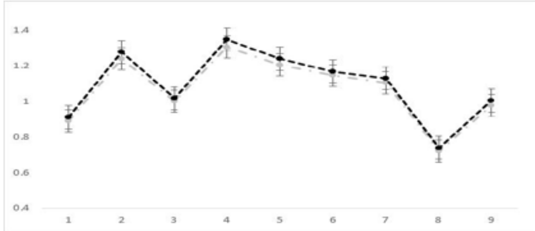

ANFO lambda=1.27, (CI=1.10-1.44) 5

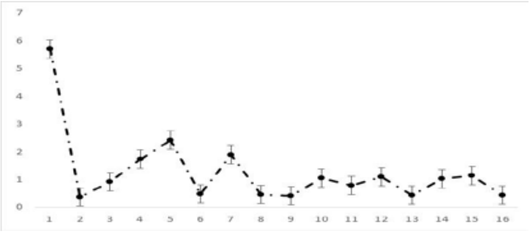

TRCR lambda=1.45, (CI=1.33-1.63) 6

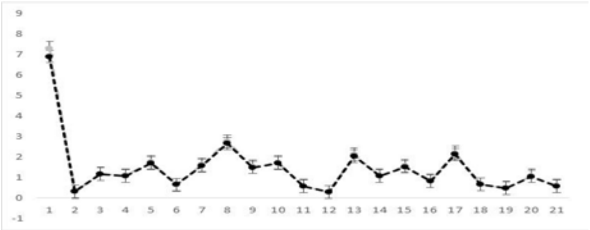

SASA lambda=0.93, (CI=0.83-1.04) 7

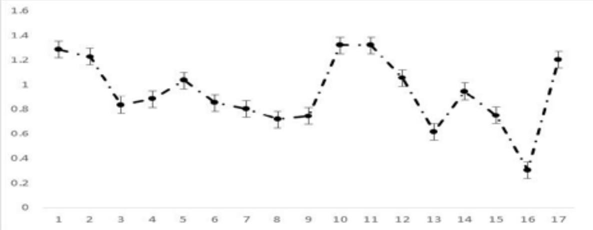

SASA lambda=1.01, (CI=0.96-1.06) 8

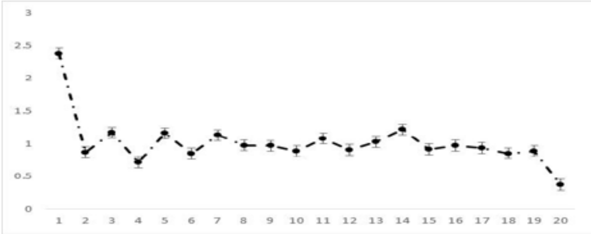

MEDITERRANEAN

Average estimated lambda values: black dashed line=males; grey line=females; X axis indicates yr in dataset. Values >1 indicate positive population growth rate; values <1 indicate negative population growth rate.

Numbers indicate datasets. Note: scale is NOT the same on all graphs. Species: TRCR – *Triturus cristatus* , RAMU – *Rana muscosa* .

TRCR lambda=1.13, (CI=1.06-1.19) 3

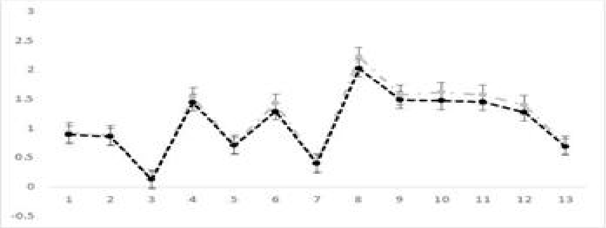

RAMU lambda=1.60, (CI=0.49-2.69) 9

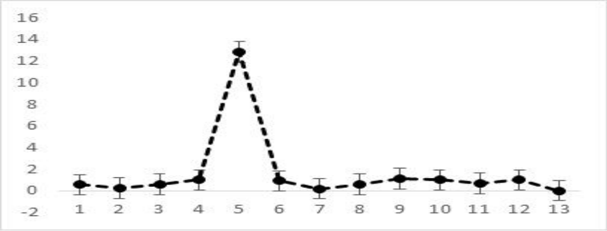

RAMU lambda=1.14, (CI=0.79-1.49) 12

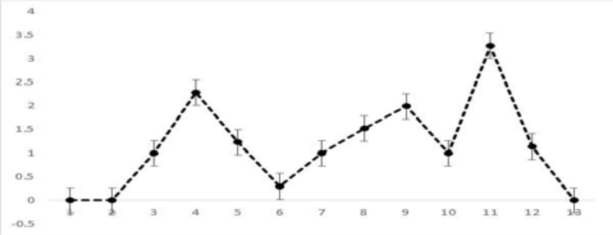

RAMU lambda=0.86, (CI=0.56-1.15) 13

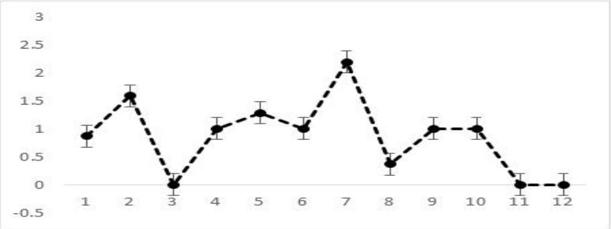

RAMU lambda=1.55, (CI=1.13-1.96) 14

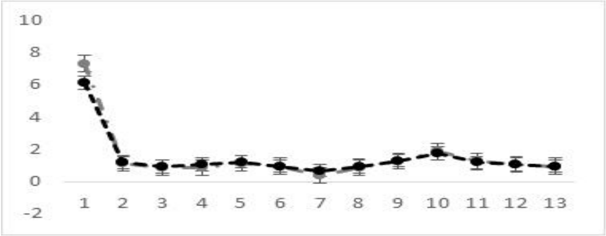

RAMU lambda=1.50, (CI=1.09-1.91) 15

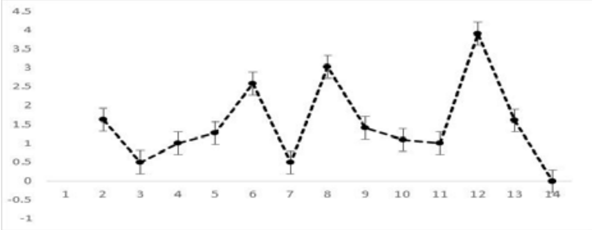

RAMU lambda=1.10, (CI=0.95-1.25) 17

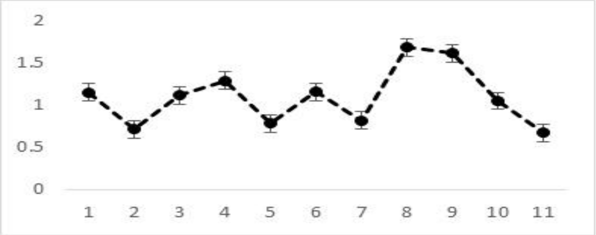

RAMU lambda=1.20, (CI=0.93-1.47) 18

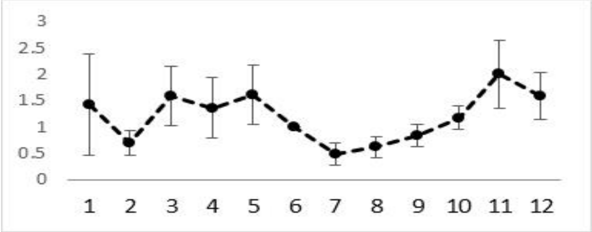

RAMU lambda=1.31, (CI=1.20-1.41) 19

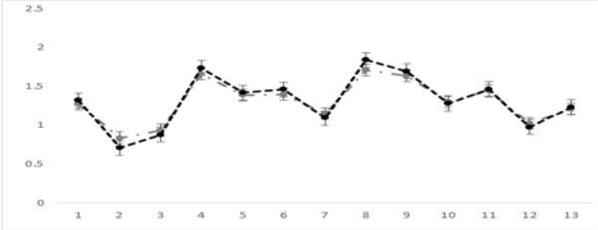

MONTANE

Average estimated lambda values: black dashed line=males; grey line=females; X axis indicates yr in dataset. Values >1 indicate positive population growth rate; values <1 indicate negative population growth rate.

Numbers indicate datasets. Note: scale is NOT the same on all graphs. Species: ANBO – *Anaxyrus boreas* , PSMA –*P seudacris maculata* , RACA – *Rana cascadae* , RALU – *R. lutieventris* , RASI – *R. Sierrae* .

ANBO lambda=0.98, (CI=0.89-1.07) 10

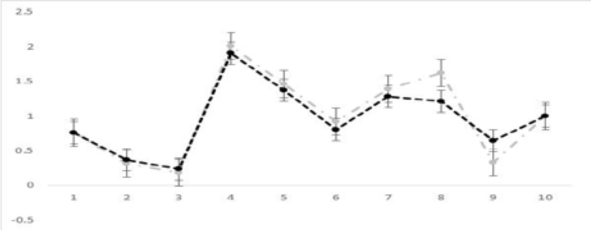

RACA lambda=1.07, (CI=1.03-1.12) 11

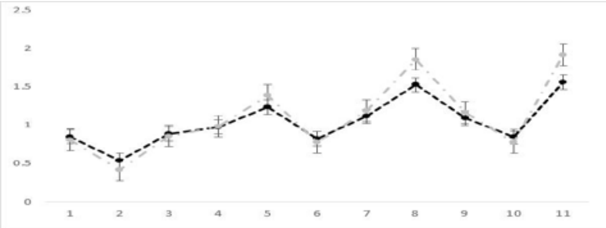

RALU lambda=1.30, (CI=1.24-1.35) 16

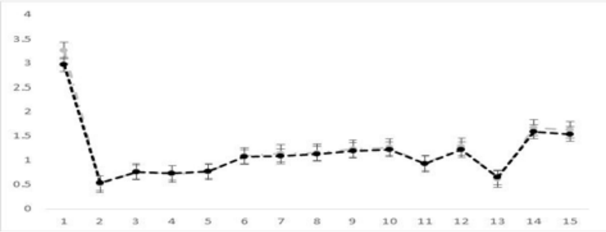

RALU lambda=1.19, (CI=1.08-1.30) 20

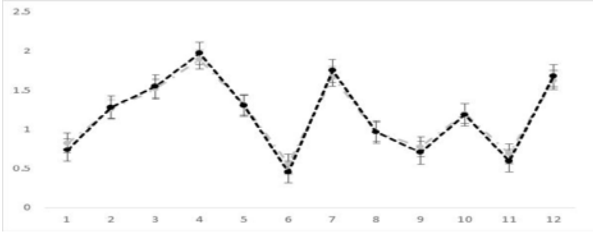

ANBO lambda=1.12, (CI=1.06-1.18) 21

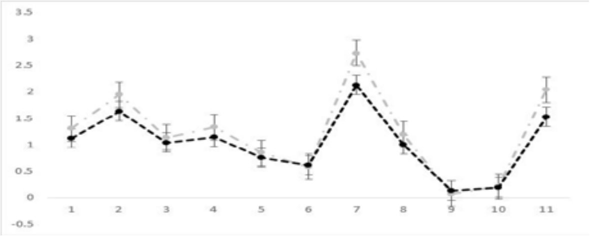

RACA lambda=1.04, (CI=0.97-1.12) 22

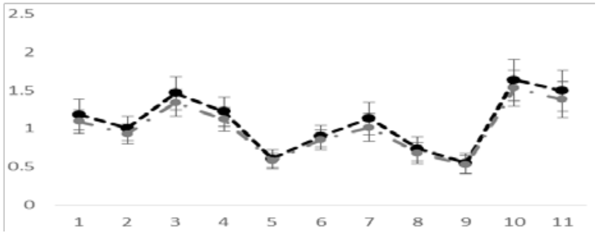

RALU lambda=0.98, (CI=0.95-1.01) 23

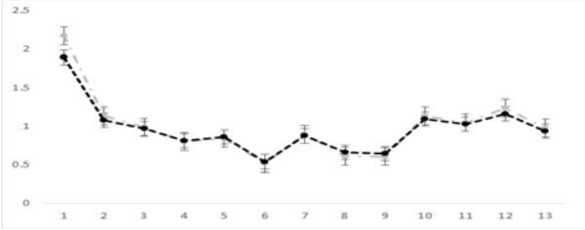

RASI lambda=1.33, (CI=1.27-1.39) 24

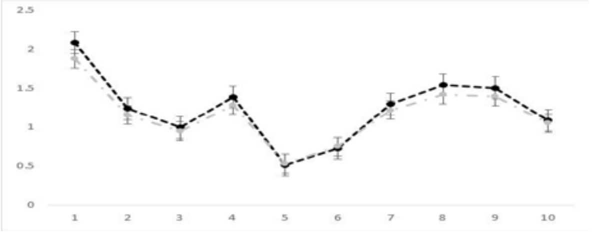

RALU lambda=1.21, (CI=1.18-1.24) 25

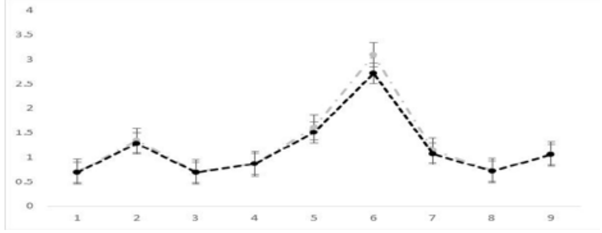

PSMA lambda=1.17, (CI=1.10-1.25) 26

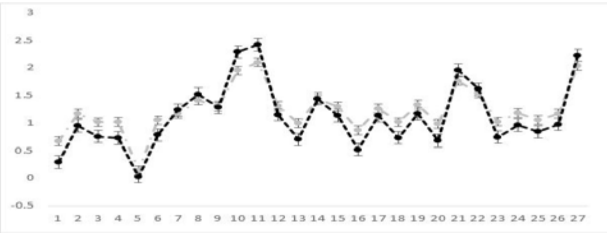

ANBO lambda=2.43, (CI=1.16-3.69) 27

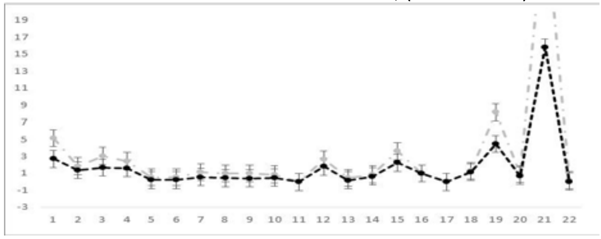

ANBO lambda=0.94, (CI=0.99-1.00) 28

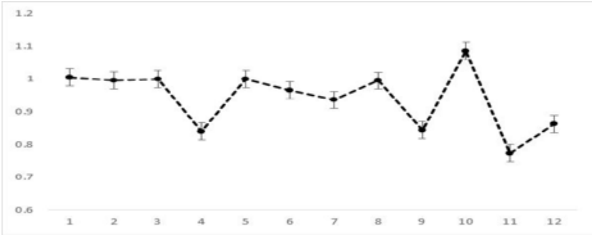

PSMA lambda=1.20, (CI=1.13-1.25) 29

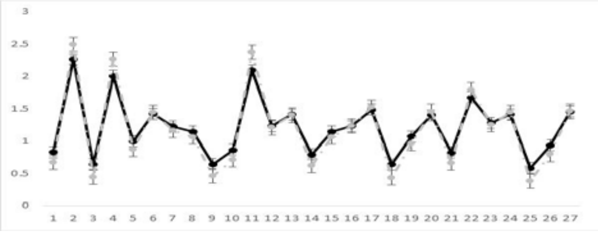

ANBO lambda=0.94, (CI=0.89-0.99) 30

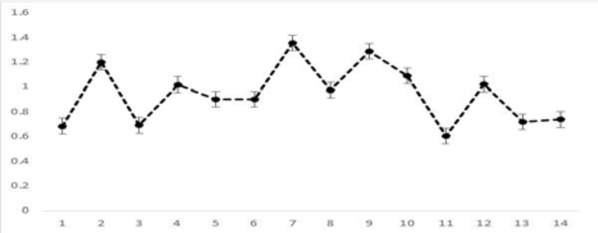

ANBO lambda=1.07, (CI=0.84-1.30) 31

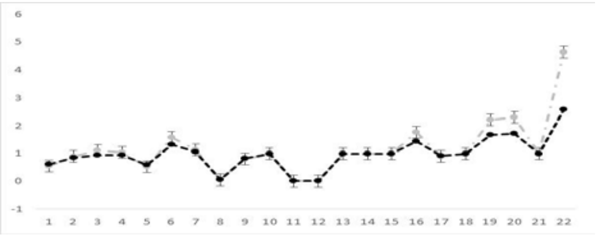

Supplement: Supplementary file 1 — Supplementary online material [file 41598_2017_17105_MOESM1_ESM.pdf]
